# Advances in MoS_2_-Based Biosensors: From Material Fabrication and Characterization to Biomedical, Environmental, and Industrial Applications

**DOI:** 10.3390/bios15060371

**Published:** 2025-06-10

**Authors:** Chun-Liang Lai, Arvind Mukundan, Riya Karmakar, Roopmeet Kaur, Kuo-Liang Huang, Hsiang-Chen Wang

**Affiliations:** 1 Division of Pulmonology and Critical Care, Department of Internal Medicine, Dalin Tzu Chi Hospital, Buddhist Tzu Chi Medical Foundation, No. 2, Minsheng Road, Dalin, Chiayi City 62247, Taiwan; 2 School of Medicine, Tzu Chi University, 701 Zhongyang Road, Sec. 3, Hualien 97004, Taiwan; 3 Department of Mechanical Engineering, National Chung Cheng University, 168, University Road, Min Hsiung, Chiayi City 62102, Taiwan; d09420003@ccu.edu.tw (A.M.);; 4 Department of Electronics and Communication Engineering, Chandigarh University, NH-05, Ludhiana 140413, India; 5 Division of Chest Medicine, Kaohsiung Armed Forces General Hospital, 2, Zhongzheng 1st Road, Kaohsiung City 80284, Taiwan

**Keywords:** MoS_2_, mechanical exfoliation, chemical exfoliation, liquid-phase exfoliation, physical vapor deposition, chemical vapor deposition, biosensor

## Abstract

The growing demand for low-cost biosensors has stimulated the study of new technologies and materials like molybdenum disulfide (MoS_2_). Due to its electroconductive nature and high surface-to-volume ratio, it allows for the ultra-sensitive detection of biomarkers. The crystal structure of MoS_2_ provides it with a unique micrometer thickness, making it appropriate for biosensing in healthcare, environmental monitoring, and food safety. As compared to traditional materials, MoS_2_ can work without labels (through field-effect transduction or plasmonic shifts) while maintaining biocompatibility and low-cost fabrication, which fill significant voids in the early diagnosis of diseases. This paper provides an overview of the recent advancements in MoS_2_-based biosensors, which are primarily focused on the field-effect transistors and surface plasmon resonance techniques and fabrication methods for MoS_2_-based biosensors like mechanical exfoliation, liquid-phase exfoliation, physical vapor deposition, chemical vapor deposition, and chemical exfoliation, applications in various industries, and their characterization techniques to evaluate the quality and functionality of MoS_2_ nanosheets in biosensors. While certain challenges remain like improving conductivity and scalability, MoS_2_-based biosensors serve as a powerful tool for the precise and reliable detection of biomarkers in environmental, food, and healthcare industries.

## 1. Introduction

The advancement of biosensing technologies has been considered a substantial growth in various industries like healthcare, environment, and the food industry, and for the early-stage detection and prevention of diseases. The increase in age-related problems like Down syndrome, diabetes, and preeclampsia in mothers, which are highly related to higher health risks for abnormalities, leads to the necessity of several diagnostic tests for early detection [1]. Early and effective detection of pathogens and infectious agents that cause diseases is essential to ensure human health safety [2]. For instance, the second most common reason for men’s death is cancer; more than 1.3 million men go through prostate-specific antigen (PSA) blood serum testing, a new prostate cancer diagnosis which is held every year. Though PSA testing has very low specificity and high false result rates, it is important to identify new biomarkers that can help to detect prostate cancer in the early stage accurately. Some of the recent developments made in the transition metal dichalcogenide (TMDC), particularly MoS_2_, led to the measurement of biomarkers with high sensitivity and specificity at a low cost of detection, employing both transistor-based and surface plasmon resonance (SPR)-based biosensor approaches, which will be distinctly outlined in this introduction [3]. Various techniques using two-dimensional biosensors for detecting the bacteria are mentioned in the below study. One of the most powerful and widely used techniques for biosensing and molecular interaction studies is surface plasmon resonance (SPR), which relies on the interaction between the light and metal surface under certain conditions [4]. It is used for the instantaneous identification of various biomarkers without requiring labels. MoS_2_ is a TMDC that has had a significant role in recent years because of its unique properties, making it ideal for biosensing applications. MoS_2_ belongs to the class of 2D materials in which weak Van der Waals forces bind different individuals exhibiting optoelectrical properties [5]. It consists of a layered architecture, which is composed of a molybdenum atom embedded between two sulfur atoms. MoS_2_ is known to have a high surface area, which leads to strong interaction with the biomolecules and serves as an important factor in enhancing the sensitivity of biosensors [6]. The unique features of MoS_2_ make it suitable for field-effect transistor-based biosensor applications and SPR-based biosensing, enhancing its versatility. The MoS_2_ architecture in these devices leads to the reduction in scattering effects and helps in improving the signal consistency, which is a major challenge faced in traditional methods. The unique features of MoS_2_ make it suitable for both field-effect transistor-based biosensor applications, reducing scattering effects and improving signal consistency, and SPR-based biosensing, which has led to significant growth compared to traditional methods [7]. MoS_2_ has high sensitivity and helps in the label-free detection of various diseases [8]. Particularly, MoS_2_ is used as a channel material to detect low concentrations of biological analytes. The biocompatibility of MoS_2_ having a high surface area allows efficient functionalization and enhancement of biomolecules with the target substances. Technological advancements made in the MoS_2_ field showed promising results in detecting disease biomarkers [9]. The exceptional properties of MoS_2_ to detect biomolecules make it suitable for medical, environmental, and food industry applications. However, the MoS_2_-based biosensors face some problems like restacking and electrical conductivity, which can also be prevented with the help of dopants like conducting polymers [10]. With an increase in research studies about MoS_2_-based biosensor studies, their potential in diverse fields including healthcare, environmental monitoring, and food safety tends to increase and provide a powerful platform for precise detection methods [11]. MoS_2_-based biosensors are fabricated using various techniques like mechanical exfoliation, LPE, CVD, chemical exfoliation, and PVD and are characterized using different methods like Raman spectroscopy, optical spectroscopy, transmission electron microscope (TEM), scanning electron microscopy (SEM), and atomic force microscopy (AFM), which are discussed below [12]. This study focuses on the recent advancements in high-performance biosensing tools and provides a comprehensive overview of the fabrication techniques, applications, and characterization techniques of two-dimensional MoS_2_-based materials with various substrates.

## 2. Materials and Methods

### 2.1. Mechanical Exfoliation

Mechanical exfoliation, which is often termed the Scotch tape method, is a technique used to produce thin layers of the materials from their bulk crystals [13]. It is one of the most powerful and efficient ways to produce the cleanest, highly crystalline, and atomically thin high-quality nanosheets of layered MoS_2_ [14]. The main purpose of this method is to weaken the Van der Waals forces between the nanomaterial layers to peel the layers apart with minimal effort [15].

**Figure 1:**
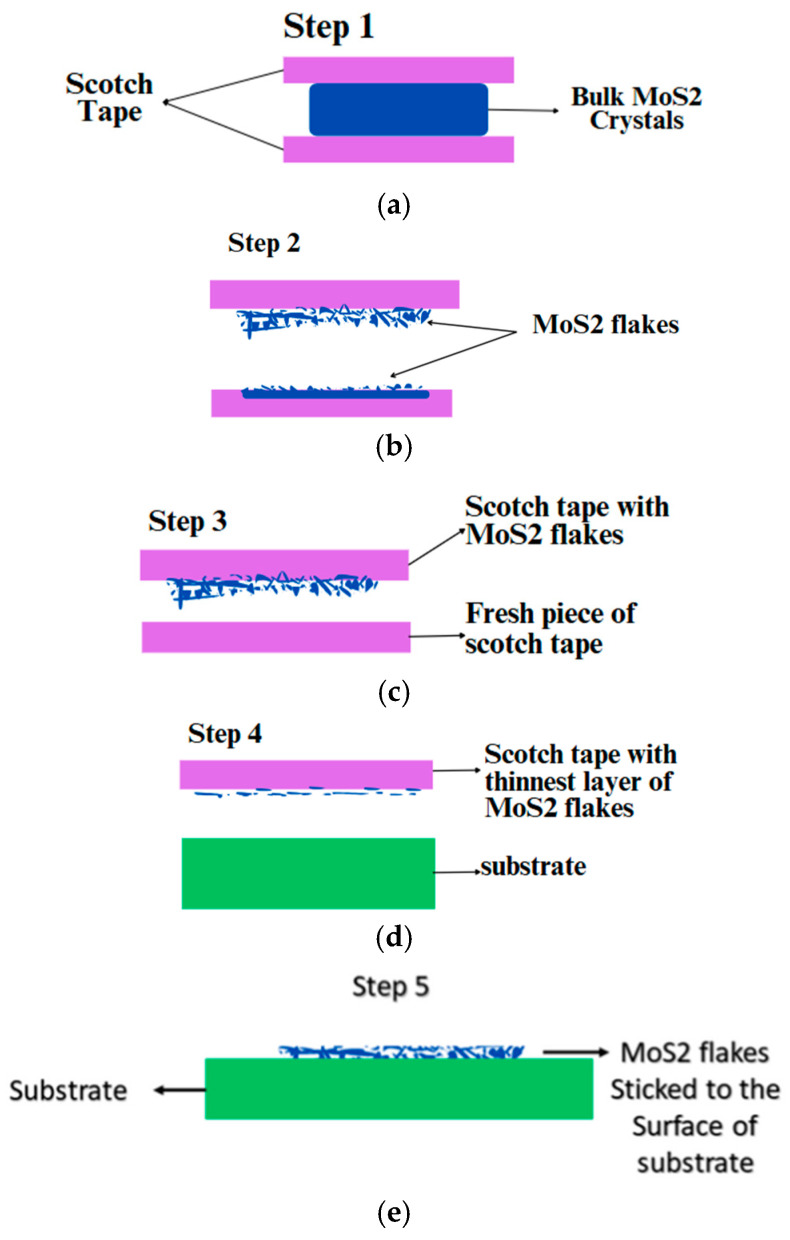
(**a**) Bulk crystals are stuck between two pieces of Scotch tape; (**b**) Scotch tape is peeled off carefully, leaving flakes behind on both pieces of tape; (**c**) Another fresh piece of Scotch tape is taken; (**d**) The Scotch tape obtained at the end with thinnest layer of flakes is stuck to the substrate and (**e**) flakes are stuck to the surface of substrate.

In this process, high-quality bulk MoS_2_ crystals are selected and Scotch tape is used to peel thin layers from the bulk crystals as shown in Figure 1a. Now, the bulk MoS_2_ is stuck between two pieces of Scotch tape for a few seconds and then peeled off carefully to avoid torn or uneven layers. The Scotch tape consisting of MoS_2_ flakes is further folded and peeled off multiple times, and the process is repeated using fresh pieces of tape to obtain the thinnest layer possible as shown in Figure 1b. The next step involves transferring the exfoliated MoS_2_ layers from the Scotch tape to the substrate by simply pressing the Scotch tape containing MoS_2_ flakes onto the substrate and peeling it off carefully, leaving the MoS_2_ layers adhered to the substrate surface [16]. Next, the substrate undergoes a cleaning process as there are chances of some residues from the Scotch tape when the flakes of MoS_2_ were transferred on the surface of a substrate; and for cleaning, mild solvents like acetone are used, followed by rinsing with deionized water [17]. After cleaning, the substrate is dried using gentle heating. Further, the exfoliated layer formed is examined according to the required thickness, uniformity, and quality of layers [18].

A method to estimate the effectiveness of peeling off two-dimensional nanosheets from the substrate without causing any fractures was proposed by Gao et al., which aimed to upgrade the peeling procedure for peak performance and better supply [19]. Their theoretical models were validated by comparing them using the coarse-grained molecular dynamics (CGMDs) simulation results. The results concluded that peeling angle and adhesive strength are the two most important terms for controlling the process of peeling. Huang et al. suggested a technique for assisting the exfoliation of monolayered MoS_2_ from their bulk crystals, and it was mainly effective where exfoliation is difficult using conventional methods [20]. Another method was developed by Hossain et al., who aimed to develop label-free identification of cancer using mechanical exfoliation methods [21]. Bulk crystals of WSe_2_, which is a layered material, are taken, which also makes it possible to separate these layers mechanically, which are later transferred to silicon or silicon dioxide substrates. Various techniques were used to ensure the nature and thickness of the exfoliated WSe_2_ layers. Another efficient method was provided by Sozen et al. for developing thin nanosheets of MoS_2_, using a unique rollup approach that creates excellent flake density [22]. This nanosheet fabrication method presents a favorable approach for the budget-friendly fabrication of ultrathin nanosheets while maintaining good optoelectronic properties. Krawczyk et al. proposed the Scotch tape method to mechanically exfoliate a thin layer of MoS_2_ flakes from bulk MoS_2_ crystals and further transfer the flakes on a polished and undoped Si substrate [23]. In this study, relative Elastic-peak electron Spectroscopy (EPES) measurements were used for evaluating inelastic-mean free path (IMFP) values in MoS_2_ with different surface stoichiometries, whereas two other sources of calculations including the Oswald et al. model and TPP-2M formula were also used for the comparison, and it was observed that the calculations made using EPES were in good agreement compared to the other two approaches. The SEM results showed that single crystalline MoS_2_ flakes exhibit high crystal quality and uniform thickness (see Appendix A, Table A1 for the summary of the studies of mechanical exfoliation).

The mechanical exfoliation process does not involve any kind of chemicals or chemical reactions and is thus categorized as a non-destructive method. It is the simplest and most affordable approach that is capable of reducing the thickness of bulk materials into thin nanosheets. It provides the flexibility to transfer the exfoliated flakes onto a predefined substrate [24]. MoS_2_-based biosensors can also be used as a good transducing material because of their large surface area and surface-to-volume ratio [25]. It is the fastest method used for obtaining 2D materials. However, it produces flakes having random thickness and sizes, making it unsuitable for large-scale production [26]. Also, the peeling process leads to a lot of wastage of the 2D material, which gets stuck on the tape instead of being used.

### 2.2. Liquid-Phase Exfoliation (LPE)

**Figure 2:**
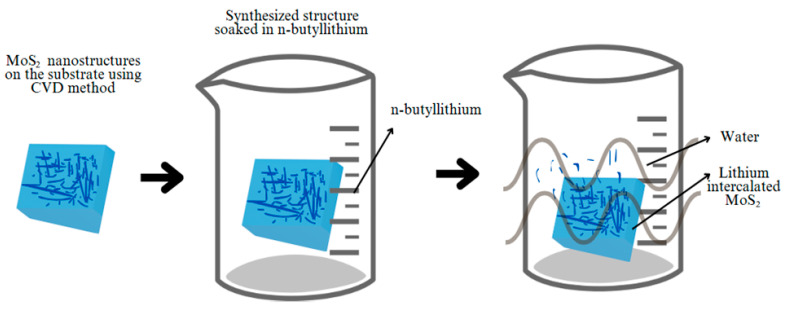
Bubbles created using ultrasonic waves are used to generate high shear forces for separating the layers from bulk material.

LPE is a technique used by dispersing bulk crystals in a liquid medium for producing 2D materials from the bulk lattice as shown in Figure 2. This method is particularly used due to its ability to produce 2D and monolayer nanosheets in bulk in a low-cost and scalable manner [27].

The first step is to select a solvent that can effectively participate in the exfoliation process. The solvents that can be used involve N-methyl-2pyrrolidine (NMP), isopropanol, and water with surfactants [28]. The most common solvent used is NMP but it has a slow volatility rate, allowing more time for the exfoliation process to occur [29]. The second step involved in this process is ultrasonication, in which bulk MoS_2_ is dispersed in the chosen solvent and ultrasonic waves are used to create cavitation bubbles which generate high shear forces that separate the layers of the materials [30]. Further, centrifugation takes place, in which unexfoliated materials and large particles are centrifugated, and the final step involves the characterization of 2D materials using various techniques like TEM, which will be discussed in the next section of this paper.

Research by Dong et al. concentrated on developing a novel approach for oil–water separation using atomically thin MoS_2_ nanosheets with the help of liquid exfoliation [31]. It was noted that the special properties of MoS_2_-coated fabric, like exhibiting both underwater and underoil superoleophobicity, make it highly effective for industrial and environmental applications. Yin et al. aimed to develop an effective antibacterial agent by synthesizing thin sheets of MoS_2_ with the help of lysozyme-assisted LPE [32]. It was found that the method used for functionalizing the nanosheets of MoS_2_ resulted in good physiological stability. A similar study for the production and patterning of high-quality 2D materials was conducted by Witomska et al., highlighting the ability to tune the properties of two-dimensional materials by manipulating their structure at the atomic level [33]. Li et al. aimed to design a fine-tuned, antibody-free approach to investigate miR-499 with the help of MoS_2_ nanosheets combined with the CRISPR system [34] (see Appendix A, Table A2 for the summary of the studies of Liquid-Phase Exfoliation).

LPE is capable of producing large quantities of 2D materials, which makes it suitable for industrial applications [35]. It involves simple steps like sonication and centrifugation, which can be implemented on a large scale [36]. LPE is cost-effective as compared to chemical vapor deposition and micromechanical exfoliation; it does not require expensive equipment or high-energy inputs [37]. It was found that a common LPE technique, i.e., long-term ultrasonic exfoliation, can introduce defects and make it difficult to control the layer number and radial size of the exfoliated nanosheets [27]. The efficiency of exfoliation highly depends on the sonication parameters such as time, power, and the type of sonication equipment used. Despite the scalability, the yield of monolayer nanosheets can be relatively low, and significant portions of the material may remain unexfoliated, requiring further processing [38]. The main drawback of LPE, specifically when using NMP as a solvent, is the presence of residues and aggregation at the interface of the exfoliated MoS_2_ flakes. This can affect the quality and properties of the MoS_2_ films produced. In order to solve this issue, various methods, such as the combination of NMP with weaker solvents like water or ethanol, can be used that may minimize residues while keeping dispersion stable, or stepwise centrifugations can be utilized to facilitate isolation of exfoliated monolayers while separating unexfoliated bulk material. Alternatively, replacing NMP with environment-friendly solvents like cyrene offers a more suitable approach without compromising exfoliation efficiency [39].

### 2.3. Chemical Vapor Deposition (CVD)

**Figure 3:**
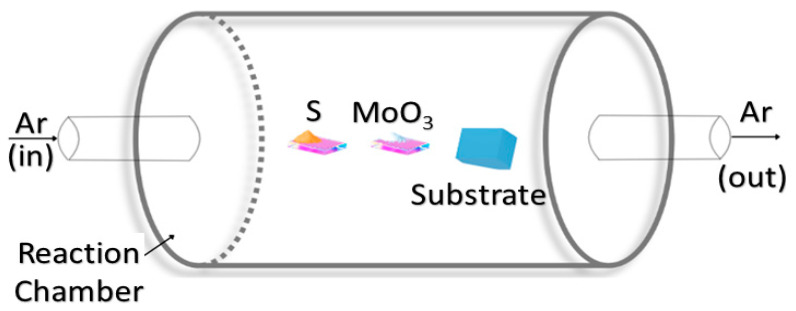
Reaction takes place between precursors sulfur and MoO_3_.

CVD is a versatile technique that produces high-quality solid materials, such as thin films of MoS_2_, by facilitating the chemical reaction of volatile precursors on a substrate surface, as illustrated in Figure 3. This method leverages controlled decomposition and deposition processes to achieve uniform and high-purity films. In this process, one or more volatile precursors, such as sulfur and molybdenum trioxide (MoO_3_), undergo a decomposition reaction or chemical reaction within a reaction chamber, depositing the desired solid material, MoS_2_, onto a substrate. This is driven by thermal energy, plasma, or other energy sources under controlled temperature and pressure conditions [40].

The first step in this process is to clean and prepare the substrate to ensure a contamination-free surface, which is essential to preserve the characteristics of the deposited film. The next step is to introduce volatile precursors like gases, vapors, or liquids which can be vaporized easily into the reaction chamber. The precursors (sulfur and MoO_3_) are transported to the substrate through the reaction chamber, carried by a carrier gas like argon, hydrogen, or nitrogen. Upon reaching the substrate, the precursors get decomposed using thermal energy, plasma, or other energy sources to form the desired solid material.

Ambient Pressure CVD (AP-CVD) was employed by Tummala et al. to synthesize thin nanosheets of molybdenum disulfide, utilizing sulfur and molybdenum trioxide (MoO_3_) as precursor materials under atmospheric pressure conditions, which simplifies the setup compared to low-pressure CVD while maintaining high-quality film growth [41]. The temperature and position of precursors are precisely controlled using a two-zone furnace setup, where distinct temperature gradients (e.g., 650 °C for MoO_3_ and 200–300 °C for sulfur) ensure optimal reaction kinetics and uniform deposition of MoS_2_ nanosheets. Their findings recorded the importance and versatility of the AP-CVD method for producing high-quality thin MoS_2_ nanosheets. Another method for the growth of thin nanosheets using CVD has been discussed by Tsigkourakos et al., in which two pieces of substrates are taken and cleaned [42]. One piece of the substrate undergoes spin-coating with Na_2_MoO_4_ solution, and the other one is kept above the first substrate. This prepared substrate is placed inside a quartz tube which is heated at 150 °C for 1 h along with sulfur in an alumina boat. This process leads to the development of MoS_2_ flakes on both substrates simultaneously. Another study to develop MoS_2_ directly on a silicon substrate was conducted by Ardahe et al. using the CVD method, which involved two main steps [43]. The first step included cleaning the substrate and placing it on the furnace along with MoO_3_ at a temperature of 850 °C. The second step involved introducing sulfur vapor at around 200–300 °C to react with MoO_3_, facilitating the sulfidation process that forms MoS_2_ layers directly on the substrate, with reaction efficiency enhanced by precise temperature control. It was recorded that at 750 °C, the films showed rectangular domains, whereas at 850 °C, the films exhibited triangular and star-shaped grains. Pondick et al. conducted a systematic investigation of CVD growth of MoS_2_, focusing on the effect of sulfur concentration, which was found to critically influence the crystallinity, layer uniformity, and defect density of the resulting films [44]. It was found that controlling sulfur vapor concentration is crucial to achieving high-quality MoS_2_ through the CVD process. In an investigation by Li et al., the researchers employed CVD to grow thin films of edge-enriched 2D MoS_2_ with a nanoplatelet network structure. The researchers were successful in tailoring the CVD parameters to create a high-porosity network of MoS_2_, where large platelets of MoS_2_ had small layered MoS_2_ sheets perpendicular to them, which significantly increased the number of edge sites within a given geometric area [45]. It was also identified that this also leads to the rise of impedance and reduced current density. Their findings highlighted the potential of their edge-enriched MoS_2_ films for catalytic applications, particularly in hydrogen production through water splitting (see Appendix A, Table A3 for the summary of the studies of Chemical Vapor Deposition).

The CVD method is widely adopted in the semiconductor industry for producing high-purity, high-performance materials, and its ability to synthesize large-area, uniform MoS_2_ films makes it particularly valuable for advanced biosensing applications. The MoS_2_ layers grown through the CVD process tend to have a higher growth rate [46]. The main advantage of this technique is the growth of several MoS_2_ nanosheets on the exfoliated montmorillonite (MMT) [47]. The most significant advantage of this method is that the same grown substrate can be used a couple of times in order to transfer the MoS_2_ layers efficiently to a specific mentioned area on the substrate [48,49]. However, it also comes with some disadvantages, i.e., controlling the layer number of MoS_2_ because according to the existing reports, the maximum monolayer area of MoS_2_ is 100 µm [50]. It also lacks in periodic growth of material, restricting the growth of MoS_2_ films on a large area [51].

### 2.4. Chemical Exfoliation

**Figure 4:**
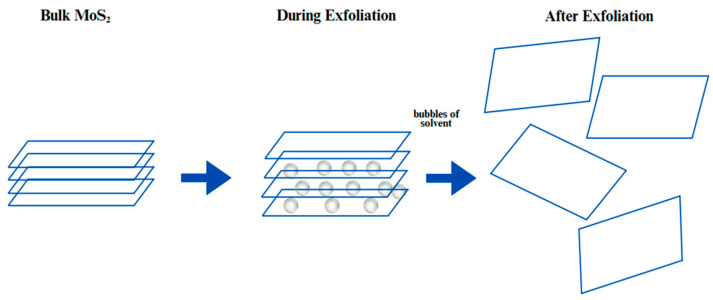
Chemical exfoliation with the help of the CVD method.

Chemical exfoliation is a technique used to convert bulk material into thin-layered nanosheets [16]. In this process, the CVD method is used, in which MoS_2_ and sulfur are taken as precursors and MoS_2_ nanostructures are grown directly on the substrate through the CVD process, which results in MoS_2_ with a high density of exposed edge sites, which is important for catalytic activity [52], as shown in Figure 4. The next step is intercalation with lithium, where the synthesized MoS_2_ nanostructures are soaked in a solution of n-butyllithium for a few hours [53]. The lithium ions present in the solution intercalate between the MoS_2_ layers, causing them to expand and weaken the Van der Waals forces that hold the layers together [54]. Further, the lithium-intercalated MoS_2_ is then exposed to water, which causes a chemical reaction where lithium reacts with H_2_O to produce H_2_ gas [55]. The hydrogen gas generated is used to exfoliate MoS_2_ into nanosheets by separating the layers.

Park et al. developed a near-infrared photodetector device with the help of chemically exfoliated multilayered MoS_2_ films, where some amount of MoS_2_ was dissolved in 1.6 M of butyllithium solution for about 48 h in a nitrogen-filled flask [56]. Further, ultrasonication was performed for about 1 h, followed by centrifugation to remove all the excess unexfoliated materials. The vacuum filtration method was used to create MoS_2_ films on a silicon substrate. An investigation by Vattikuti et al. focused on the synthetization of MoS_2_-based nanosheets used for photocatalytic applications. In this study, MoS_2_ nanosheets were synthesized with the help of a one-pot hydrothermal method by exfoliating them in a dimethylformamide solution [57]. The method presented by this study produced MoS_2_ nanosheets with enhanced photocatalytic performance, providing a promising material for environmental applications such as pollutant degradation. The performance of a flexible photodetector was investigated by Wei et al. based on a molybdenum disulfide and trioxide mixture, where powder of MoS_2_ was chemically exfoliated with the help of n-butyllithium. The researchers were successful in manufacturing the flexible photodetector using a mixture of MoS_2_ and MoS_3_, resulting in a device with high quantum efficiency [58]. Zhu et al. aimed to develop an effective method for the large-scale exfoliation of MoS_2_ nanosheets. The researchers introduced a novel organolithium reagent, pyrene lithium, which has a redox potential well-matched between the intercalation (1.13 V) and decomposition (0.55 V) potentials of bulk MoS_2_. This matching allowed precise Li^+^ intercalation without causing structural damage. The exfoliated nanosheets exhibit excellent quality and electrochemical properties, making the method highly promising for large-scale production and practical applications [30]. Sahoo et al. developed an environmentally friendly method to synthesize few-layer MoS_2_ nanosheets using acetone as a solvent, capitalizing on its unique properties as a greener alternative to NMP. Thin nanosheets were prepared from bulk MoS_2_ at various initial concentrations (0.08 mg/mL, 0.12 mg/mL, 0.3 mg/mL, and 0.4 mg/mL) using an ultrasonic bath. Although the study emphasizes the scalability of chemical exfoliation, achieving a notably higher exfoliation rate of 3.816%/h compared to conventional LPE methods, it does not clearly specify which concentration serves as the benchmark for comparison against traditional MoS_2_ preparations. Furthermore, while the process is presented as superior, no quantitative data are provided to support the performance comparison with nanosheets exfoliated using sulfur dioxide or other conventional solvents [59]. By adjusting the conditions of the chemical exfoliation process, such as the intercalation time and concentration of reactants, it is possible to regulate the dimensions and thickness of the exfoliated MoS_2_ flakes [60]. Exfoliated MoS_2_ nanosheets show significantly better performance in comparison with the bulk MoS_2_ [61]. While the yield is higher, the process still requires considerable time to achieve significant exfoliation. Achieving consistent results with chemical exfoliation is also challenging due to the sensitivity of the process to various parameters, such as temperature, concentration, and reaction time, which requires careful optimization and control (see Appendix A, Table A4 for the summary of the studies of Chemical Exfoliation).

Chemical exfoliation and CVD are two different ways of synthesizing 2D materials such as MoS_2_. CVD utilizes gas-phase precursors reacting upon a substrate at high temperatures (~700–1000 °C) to deposit high-quality, layer-by-layer films uniformly, making it suitable for scalable, device-ready applications. Chemical exfoliation, in contrast, utilizes liquid-phase reactions at low temperatures to fragment large crystals into nanosheets and produces dispersed flakes for solution-based processing at higher defect densities. CVD provides better crystallinity and thickness control, yet chemical exfoliation is faster, less expensive, and suitable for the mass production of nanocomposites.

### 2.5. Physical Vapor Deposition (PVD)

**Figure 5:**
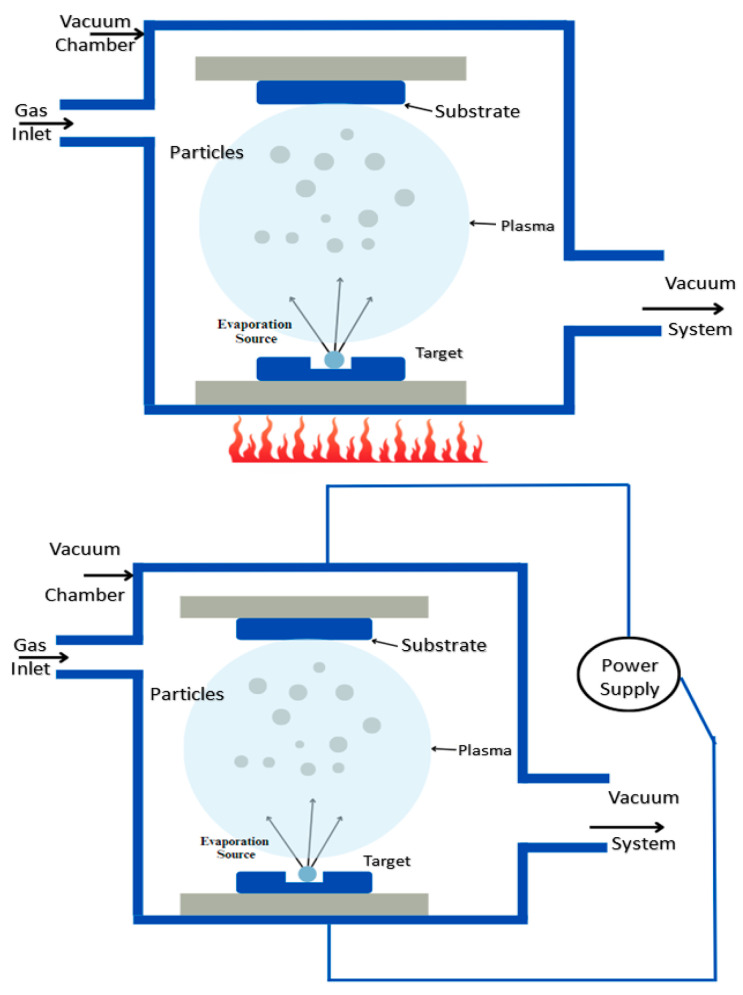
(**Top**) Evaporation-based physical vapor deposition; (**Bottom**) Sputtering-based physical vapor deposition.

PVD is another method for depositing thin films of a substance on a given substrate [62]. Based on the extraction of particles, PVD is further classified into two types of processes; for evaporation-based PVD, the material that is to be coated onto the substrate is heated until vapors are formed, whereas in sputtering-based PVD, atoms are extracted from the target material by forming ions from the plasma as shown in Figure 5 [63]. It occurs under vacuum conditions, ensuring high-purity and high-performance deposition of thin films. The initial phase in this process is cleaning the substrate to avoid contamination. Further, the vaporized atoms condense on the substrate surface, forming a thin film. In sputtering-based PVD, a high-voltage electric field, typically ranging from 200 to 1000 V, is applied to generate a plasma. The positively charged ions from the plasma bombard the target material, causing atoms to be ejected and deposited onto the substrate.

Majid et al. used PVD to synthesize copper-doped and undoped thin films of tin sulfide [64]. Their findings showed that doping the thin films of SnS with copper leads to the alteration of certain properties. In research conducted by Dong et al., the PVD process is used to enhance the wear and oxidation resistance of MoS_2_ films by introducing the nanoparticles of Cr_3_O_4_. The study enhances the tribological properties like lower friction and better wear resistance, hence making them more suitable for applications requiring lubrication in variable environments [65]. Uniyal et al. fabricated a biosensor which is based on the bimetallic layers of Ag and MoS_2_ [66]. The biosensor was fabricated on the BK7 prism base, and a layer of Ag was deposited on the BK7 base using the PVD method, followed by the formation of other layers using the CVD method. The authors revealed that the proposed biosensor tends to have much better sensitivity than the other conventional structures. Roy et al. employed a method to detect uric acid in human blood using MoS_2_ along with some nanocomposites of cobalt on a copper substrate with the help of the PVD approach [67]. It was recorded that the fabricated device has improved sensitivity, a long shelf life of about six months, and high specificity, which makes it suitable for real-time clinical applications. Ono et al. employed the PVD method to elucidate the process of film formation (see Appendix A, Table A5 for the summary of the studies of Physical Vapor Deposition). The study showed that the challenges faced in traditional methods like CVD and exfoliation were overcome by the PVD method [68].

The PVD technique is widely used in industrial applications like drilling, milling, and welding tools. This method has a controllable coating composition with an environment-friendly nature. The coatings of PVD are used as a barrier between the metal and the environment to avoid corrosion and protect the substrate’s surface [69]. The PVD setup based on a vacuum chamber is relatively cheap and includes no expensive electronic units, whereas the commercial PVD setups are very expensive and not available for small labs and student education centers [70]. This approach ensures faster coatings while maintaining the quality of the material [71]. This technique involves high temperatures for deposition to take place, which can restrict the use of substrates sensitive to high temperatures. PVD is also line-of-sight, leading to non-homogeneous coating for complex geometries or three-dimensional shapes without multiple rotations. Furthermore, PVD’s material choice is also restricted, some alloys or non-metallic compounds being hard to sputter or evaporate. Inadequate adhesion in some substrates in the absence of extensive surface pretreatment can further restrict its use.

## 3. Applications of MoS_2_-Based Biosensors

### 3.1. Cancer Detection Studies

Early detection of cancer minimizes the death rate to a great extent. MoS_2_-based sensors are highly effective in detecting and treating cancer due to their unique properties, like high surface area, as shown in Table 1 [72]. These properties of biosensors help them in adsorbing the molecules of drugs, leading to an improvement in the specificity. Optical biosensors dominate over the other biomedical instruments like enzyme-linked immunosorbent assays (ELISAs), PCR, and electrochemical sensors in detecting such diseases with high accuracy because of properties like its high sensitivity and speed of light. The speed of light property is crucial because it enables instantaneous signal transmission, allowing immediate tracking of biomarker interactions without delays. On the contrary, other instruments, such as ELISA, take hours to produce outputs, and PCR requires cumbersome sample preparation. Light–matter interactions (e.g., plasmonics and photoluminescence shifts) in optical biosensors quantify trace changes in biomarkers at high resolution and can be effectively used to diagnose cancer in its preliminary stage. These sensors are the composition of SnSe allotropes and the heterostructure of MoS_2_. One such study based on optical biosensors was conducted by Hossain et al., who investigated a five-layered SPR biosensor using MoS_2_, which is capable of identifying various cancerous cells, such as breast cancer and blood cancer [73]. SPR biosensors are based on the principle of attenuated total reflection (ATR). The sensor has five layers coupling with a prism at the end, which is reliable for wave vector matching (WVM), a crucial step for SPR condition to occur. This MoS_2_-based biosensor is typically based on the Kretschmann configuration, which is an optical setup where light passes through a prism to excite surface plasmons at a thin metal film interface. It relies on total internal reflection to generate an evanescent wave that couples with the metal’s electrons at a resonant angle and can easily detect the changes with high sensitivity. Mukundan et al. developed an MoS_2_-based biosensor to locate lung cancer cell types, leading to a significant reduction in long waiting times for cancer detection in hospitals [74]. N-type MoS_2_ were grown by the CVD process and a thin film of p-type Cu_2_O was grown. The crystal-based structure of the MoS_2_ flakes grown was examined using the TEM technique. The MoS_2_-grown flakes underwent preliminary processing, shifting the sample onto a Cu_2_O thin coating to form the overall p-n junction. Glutathione (GSH) emerged as a crucial biomarker for detecting cancer and various other diseases. A study conducted by Rawat et al. developed an MoS_2_-based electrochemical sensor that focused on the highly selective detection of GSH, which was fabricated with the help of standard semiconductor processing techniques. The Glutathione-S-Transferase (GST) was anchored on the surface of MoS_2_ and GSH was determined by the electrochemical activity of GSH and CDNB in the presence of GST. The study claimed that the MoS_2_-based sensor exhibits excellent stability and repeatability, making it suitable for cancer diagnosis and quantification [75]. Sri et al. fabricated an MoS_2_-based nanosensor to detect Tumor Necrosis Factor (TNF) in cancer patients using the electrophoretic deposition method. The biosensor was fabricated by using the electrophoretic deposition technique on a glass substrate coated with Indium Tin oxide. TNF serves as a critical factor in apoptosis and cancer and it was the first biosensor fabricated to detect TNF in cancer patients [76]. The study also proved that MoS_2_ is a favorable material for the manufacturing of this biosensor due to its 3D structure and high surface area.

An MoS_2_-based fluorescence sensor was formed by Cai et al. to find tumors in women’s breasts for the initial diagnosis [77]. The miR-21 is one of the most important biomarkers for the early-stage diagnosis of breast cancer. The fabricated device tends to have high sensitivity and efficiency, and is less expensive, thus making it favorable for miR-21 location in breast cancer patients. In this study, MoS_2_ contacts a DNA probe for fabrication, which is further followed by a DNA-miRNA hybridization process. Another study was provided by Hu et al. using an MoS_2_ biosensor based on Chalcogenide MoS_2_ Au-Ag nanocomposites having higher sensitivity and satisfactory repeatability and stability [78]. MoS_2_ was successfully synthesized and proved to be a valuable material to improve the detection performance. The peaks of silver and gold established the effective electrodeposition of metals on the MoS_2_-modified connector. The components of MoS_2_-Au-Ag were studied and analyzed using energy dispersive spectroscopy (EDS). The results of the study claimed that the modified glass carbon electrode has high sensitivity and the framework of the fabricated sensor was investigated by TEM. Peng et al. aimed to develop a highly sensitive but cost-effective approach for the detection of PD-1—a checkpoint regulator related to cancer immune evasion. The sensor was fabricated using MoS_2_ nanosheets as the quenching agent and nanofiber paper was used as a substrate for the immunosensor. The fabricated sensor is cost-effective, portable, and exhibits excellent quenching efficiency and excellent selectivity with its practical applicability in clinical applications [79]. Another study was conducted by Lai et al. using a photoelectrochemical biosensing chip based on MoS_2_, aiming to achieve better sensitivity through a straight interdigitated zigzag electrode and growing an MoS_2_ layer on a silicon-based substrate using the CVD method [80]. The experiment is mainly divided into two parts: fabrication (including three sub-parts: electrode fabrication, cell culture, and MoS_2_ growth) and measurement, where a p-type substrate of thickness about 200 µm was used. The N-type substrate was used as a base substrate on which MoS_2_ was grown. There are two primary procedures for the experiment to occur; generating the electron–hole pairs by the photoelectric effect is the initial step and the second step involves the occurrence of an electrochemical reaction between the electron–hole pairs and samples produced by the photoelectric effect. Research was also conducted to examine the correlation between the number of modified cells and the photocurrent. Qin et al. conducted a study to identify several biomarkers by employing MoS_2_ as a tunable electrochemical biosensor. The feature of MoS_2_ to stimulate the enzymes for changing the substrate color is used to determine cancer biomarkers. The study also claimed that the process involved is low-cost, simple, and has high sensitivity and faster speed as compared to the other traditional methods used for cancer biomarker detection [81]. Another study for tumor therapy was conducted by Cao et al., aiming to develop a multifunctional nanoplatform for targeted cancer therapy. The platform developed in the study consisted of upconversion nanoparticles combined with MoS_2_ quantum dots. The motive was to develop multifunctional nanoplatforms for improving the precision and efficacy of cancer treatments by minimizing the side effects [82]. Enzyme-based nanosensors like ZIF@HAgel-GOx leverage metabolic pathways for cancer therapy, but MoS_2_ biosensors offer superior capabilities. Unlike enzyme systems that are limited by stability issues and specific metabolic targets, MoS_2_ biosensors provide exceptional versatility, enabling ultrasensitive, multimodal detection of diverse cancer biomarkers through electrical, optical, and plasmonic sensing mechanisms.

The integration of MoS_2_ in biosensing technology constitutes a revolutionary step in early cancer detection, providing an exclusive combination of chemical, electrical, and optical properties that make it immensely appealing for use in biomedical applications. One of the most significant benefits lies in its high surface area-to-volume ratio, which supports high drug-loading rates with better interaction with biomolecules. This aspect ensures that even trace levels of cancer biomarkers can be easily captured and sensed, which is pivotal for early detection. Apart from this, MoS_2_ also boasts exceptional photoluminescence and plasmonics, which make it an attractive option for optical biosensors that demand high sensitivity and real-time detection features. Its tunable electronic bandgap also enables scientists to design it for different configurations of sensors, i.e., electrochemical, fluorescence, or photoelectrochemical biosensors. While these attributes make MoS_2_ an exciting material for use in sensors, it is equally important to examine its limitations. One of its major drawbacks is that it is generally difficult to produce reproducible and homogeneous synthesis of MoS_2_ nanosheets, which was achieved by taking up methodologies such as CVD. The thickness of layers, direction of crystalline structure, or purity differences may lead to deviations in the performance of sensors. Other than that, although MoS_2_ allows for good biocompatibility, minimal study of long-term toxicity behaviors and biodegradation has been carried out, which causes concern for its safe integration with medical practices. Apart from that, though MoS_2_ is renowned for multifunctional sensory functionality, the integration of these capabilities in a miniaturized, portable, and affordable system still remains an outstanding problem in design.

### 3.2. Medical Industry Studies

MoS_2_ has various applications in the field of the medical industry and employing MoS_2_-based sensors in the medical field instead of traditional methods is rapidly increasing due to its distinct properties like biocompatibility, high sensitivity, and selectivity as shown in Table 2 [83]. A study by Rehman et al. investigated dual modification of h-MoS_2_ nanosheets with electrodeposited nickel nanoparticles (NiNPs) for simultaneous biosensing of uric acid (UA) and dopamine (DA). They highlighted an easy ultrasonic method of synthesis of h-MoS_2_ from a bulk of MoS_2_ with subsequent electrodeposition of NiNPs over glassy carbon electrode (GCE). The prepared GCE electrochemical sensor showed high sensitivity and selectivity, attributed to synergistic effects between 2D material and metal nanoparticles. Cyclic voltammetry (CV), differential pulse voltammetry (DPV), and electrochemical impedance spectroscopy (EIS) characterization confirmed improved sensing performance. Low detection limits of 7.3 µM and 2.1 µM for UA and DA, respectively, were achieved by the sensor, with impressive anti-interference capability even in the presence of interfering substances like glucose and ascorbic acid. Thus, it serves as a promising small-molecule detection platform for use in clinical diagnosis [84]. Another study conducted by Campos et al. investigated the MoS_2_ nanomaterials exhibiting excellent absorption in the near-infrared window, making them suitable for controlled drug release, which enhances the accumulation of drugs at the tumor site by minimizing the side effects on the healthy tissues [85]. The preparation of MoS_2_-based nanosheets was completed using the LPE method, followed by intercalation with PVP. Further, ultrasonication was performed for about 6 h, ensuring effective penetration and interaction with cells for retaining necessary photothermal properties. Nurrohman et al. claimed that MoS_2_-based biosensors are highly effective in photothermal therapy where near-infrared light is converted to heat for inducing localized thermal ablation of cancer cells and tissues. These sensors are also capable of detecting biological molecules and other analytes, making them valuable for medical industries [86]. Most diseases progress due to changes in biomolecules such as proteins, DNA, and uric acid, which require timely detection for effective diagnosis and treatment. This early recognition is possible with the help of MoS_2_-based biosensors. Meng et al. discussed the progress that has been made in TMDCs for medical examinations, detection of enzymes, and other biological molecules. In this method, a special CVD method has been applied in the synthesization of two-dimensional TMDCs, where the precursors are passed through a furnace as a gas and react at a very high temperature with the carrier gas to form a substrate layer [87].

The DNA of the chikungunya virus was detected by Singhal et al. using thin-layered MoS_2_ nanosheets based on disposable biosensors. The researchers claimed that the study is the first electrochemical detection approach for detecting chikungunya virus DNA. The proposed biosensor exhibited a maximum range of 100 µm and a minimum range of 0.1 nm towards the chikungunya virus DNA. The sensor was fabricated using screen-print terminals, which are proficient for the probe DNA binding. The fabricated sensor possesses unique properties like high linearity and economic feasibility which make it useful for miniature applications. The comparative study showed that the response of PDNA is nearly equal to that of non-complimentary DNA [88]. Liu et al. proposed a study to develop a highly sensitive field-effect transistor (FET) using MoS_2_ films for identifying trisomy 21 syndrome DNA. The CVD technique was used to grow the MoS_2_ film on the silicon or silicon dioxide substrate. The researchers claimed that the developed biosensor is applicable because of its low cost and high sensitivity, and that it is suitable for large-scale applications in the medical industry. The fabricated sensor could detect DNA even at extremely low concentrations, with detection limits under 100 aM, and provides a high response rate with high sensitivity and high specificity in detecting DNA sequences, making the sensor effective for Down syndrome detection. This approach offers an early, accurate, and cost-effective alternative to the traditional methods used [89]. Wen et al. fabricated and optimized an MoS_2_-based FET biosensor for the detection of Tau protein, which is very crucial for the preliminary detection of Alzheimer’s disease. The detection of Tau protein in blood is challenging with traditional methods and thus requires a novel, sensitive detection method. This study aims to provide four different biofunctionalization methods to enhance the performance of biosensors: No linking molecule (direct adsorption of antibodies on MoS_2_ surfaces), PASE-stacking (PASE is used as a linking molecule bound to the surface of MoS_2_), APTES-GA covalent bonding (covalent bonds are formed between the surfaces of MoS_2_), and Oxygen Plasma Pretreatment (MoS_2_ is pretreated with oxygen plasma). The nanofilms of MoS_2_ produced by the mechanical exfoliation technique were deposited on the silicon substrate. The performance of the biosensor was dependent on the four different methods of biofunctionalization [90]. A label-free electrochemical biosensor was established by Raj et al., aiming to identify escherichia coli. The hydrothermal technique was used for the synthesization of MoS_2_ by combining it with PANI using in situ oxidative polymerization and fabricating the sensor on the glassy carbon electrode [91]. The sensor was fabricated by combining AuNPs, PANI, and MoS_2_ using the green hydrothermal method, followed by situ oxidative polymerization for enhancing the electrical conductivity and surface area, and the chemical reduction method to improve the sensitivity. The fabricated sensor was practically tested by analyzing the urine samples for clinical use and serving public health and food safety. The study claimed that the sensor exhibited unique properties with low detection limits, making it a promising material for early detection of the pathogen in clinical, food, and environmental monitoring. Chen et al. aimed to develop an MoS_2_-based microfluid biosensor for the immune detection of Toxoplasma gondii, a zoonotic parasite. The developed biosensor showed excellent properties, proving its potential applications in biomedical fields to detect toxoplasma gondii, and having a good detection range [92]. The researchers claimed that the novel approach to integrate TCMF with MoS_2_ enhanced the biosensing performance of the sensor and the sensor’s specificity was also confirmed against other viruses. García et al. provided a study highlighting different nanomaterials and their potential applications in advanced sensing technologies. The study focused on the development of water purification systems for the removal of pollutants, detection of food contaminants, and monitoring of crop health [93]. However, while silicon nanowire (SiNW) field-effect transistors are widely used for label-free detection of biomarkers, MoS_2_-based biosensors demonstrate superior performance in medical diagnostics. Unlike SiNWs, which suffer from surface oxidation and limited sensitivity in physiological fluids, MoS_2_’s atomic thickness and high surface-to-volume ratio enable the detection of biomarkers even in complex biofluids.

MoS_2_ has become an effective and multifunctional material for medical diagnostics and therapeutics due to its inherent physicochemical properties, particularly because it is biocompatible and atomically layered, with an extremely large surface area. It is an optimum material to interact with biomolecules such as DNA, proteins, enzymes, and small-sized metabolites. One of the greatest properties of MoS_2_ sensors is that they are extremely sensitive and selective, which allows for ultra-low disease biomarker detection. This is particularly important for early detection, especially in complex diseases like cancer and Alzheimer’s disease, or genetic diseases like trisomy 21. FET and electrochemical sensors based on MoS_2_ have exhibited steady performance in biomarker detection at sub-attomolar levels, a sensitivity that conventional techniques hardly attain. The tunability of MoS_2_ also facilitates functionalization with selected targets so that MoS_2_ can be a highly versatile sensor material. Processes like CVD and exfoliation provide fairly controlled synthesis routes for MoS_2_ with resulting thin films and nanosheets suitable for integration with devices. Optical properties like significant near-infrared absorption make MoS_2_ suitable for photothermal cancer therapy and drug delivery with controlled release. Photo-heat conversion capability permits ablation of cancer tissue by concentrating heat without damaging normal cells, eliminating chemotherapy- or radiation-induced side effects. These advantageous properties are compounded by affordability and bendable substrate compatibility, all good qualities that are appropriate for point-of-care and wearability diagnostics. These advantageous properties, however, have some drawbacks. One such drawback is synthesis and functionalization. While high-quality MoS_2_ can be supplied by CVD, the process is not optimized for economy or for scaling up to large production.

### 3.3. Food Industry Studies

The development and advancements of MoS_2_-based biosensors have led to various emerging applications in food and bioscience as shown in Table 3. Li et al. provided a study on the importance of biosensors based on two-dimensional materials for food safety. It was noticed that nitrite content was rapidly increasing in crops because of an increase in inorganic fertilizers sprayed in the fields. However, the nitrite content tends to enter the human body along with the food products, which converts the hemoglobin into methemoglobin and hence disrupts the oxygen delivery system in the human body, making it crucial to detect and control the nitrite content effectively. Therefore, two-dimensional-based biosensors play a vital role in food safety and biomolecular detection [94]. Another study for the detection of synthetic colors in food samples was conducted by Jahani et al., aiming to reduce the side effects of artificial colors in humans. The main goal of this study was to synthesize MoS_2_-based biosensors for modifying the electrodes to identify sunset yellow FCF synthetic food dye in food samples, and this MoS_2_-based biosensor was successfully implemented to detect FCF in real-life samples [95]. Nehru et al. proposed an MoS_2_-based sensor grown on g-C_3_N_4_ nanotubes to determine vanillin (VNN) in food samples using the hydrothermal method. Vanillin is an artificial flavoring agent used in perfumes, food products, and beverages, and is beneficial when used in limited quantities. However, the overdose of VN is hazardous and needs to be detected using a cost-effective g-C_3_N_4_-grown nanotubes approach. The proposed method used for the detection of vanillin in food samples proves to be a very cost-effective and more attractive strategy for food additive detection [96]. Lin et al. demonstrated an approach for detecting terbutaline sulfate in food samples that were characterized later using techniques like TEM and SEM. The researchers used fresh samples of pork for testing and claimed that the fabricated sensor had excellent electrolytic properties [97]. Another MoS_2_-based user-friendly sensor was developed by Sharma et al. to detect Xanthine (Xn) in fish meat. Keeping fish meat fresh and free from bacteria like Vibrio and Salmonella is a major concern as fish meat has a large market in various industries like pharmaceuticals for multiple medications or fish oil supplements [98]. Xn is one such bacteria found in rotten fish and the level of Xn increases with an increase in the storage time of fish meat, making it necessary to keep track of Xn in fish meat for diagnosing and monitoring diseases.

Govindasamy et al. proposed an approach where carbon nanotubes are coated with MoS_2_ for detecting CAP in food samples like milk [99]. There are various techniques used for the monitoring of CAP levels in food items but electrochemical methods are low-cost, simple, and portable as compared to other traditional analytical approaches. Another study using carbon nanotubes based on two-dimensional MoS_2_ was conducted by Cheng et al. to detect Thiabendazole (TBZ) residues in food samples [100]. The sensor was fabricated using multiwall carbon nanotubes aligned with layered MoS_2_ nanosheets. Zhou et al. demonstrated the use of MoS_2_-based electrochemiluminescence (ECL) biosensors for ensuring food safety by detecting contaminants in various samples. There are different types of ECL biosensors available for food sensing applications and all are well known for their high sensitivity, selectivity, and efficiency, especially for examining miniature devices. This approach for sensing the food samples is cost-effective and biocompatible [101]. Bankole et al. focused on developing biosensor technology mainly in the food and healthcare industry. The study used novel nanomaterials which also improve the sensitivity, selectivity, and stability of the biosensors [102]. A photoelectrochemical immunosensor based on MoS_2_ nanocomposites was developed by Ai et al., aiming to detect N6-methyladenosine, an essential RNA modification. The biosensor was fabricated by assembling an MoS_2_ heterojunction, gold nanoparticles, and an m6A antibody. The main aim of this study was to improve the sensitivity and selectivity of the biosensor, hence making it suitable for various environmental and biological applications [103]. While ELISAs remain the gold standard for detecting food contaminants like allergens and pathogens, MoS_2_-based biosensors offer significant advancements for food safety monitoring. Unlike ELISA systems that require lengthy incubation times (typically 2–4 h), complex antibody handling, and specialized lab equipment, MoS_2_ biosensors enable rapid on-site detection through their exceptional electrical and optical properties.

MoS_2_-based biosensors have opened up new doors for food safety assurance, product-quality surveillance, and biomolecular identification in food and bioscience industries. With exceptional surface area, catalysis, and tunable bandgap properties, MoS_2_ is particularly suitable for electrochemical and optical sensing. One of its main merits is that it can identify toxic additives, synthetic food colors, drug residues, and indicators of spoilage with sensitive detection and high selectivity. For example, Li et al. highlighted the imperative to track nitrite content in food due to its toxic conversion to methemoglobin in humans. MoS_2_-based sensors provide a fast, efficient solution to this end, providing better response times with minimal operating complexity compared to traditional approaches. Further, scientists like Jahani et al. and Nehru et al. have illustrated MoS_2_’s capability to identify food coloring such as sunset yellow FCF and flavoring chemicals such as vanillin with affordable probing using inexpensive sensing substrates. These sensors provide significant substitutes for costly spectrophotometric or chromatographic practices that are not only time-consuming but also require complex instrumentation. Further integration of MoS_2_ with hybrid nanocomposites from material such as carbon nanotubes, g-C_3_N_4_, and Au nanoparticles helped improve not only sensor stability but also electrical conductivity, and transduction of the signal. These improvements are visible in works such as those of Govindasamy et al. and Cheng et al., in which MoS_2_-based nanocomposites identified CAP, thiabendazole in milk products, and other foods. Yet another strong point of MoS_2_ in food surveillance is that it can be applied to a range of detection modes such as electrochemical, photoelectrochemical, electrochemiluminescence (ECL), etc. Illustrating this with an example from Zhou et al., ECL-based MoS_2_ sensors possess high sensitivity and reproducibility, which are critical in detecting contaminants in trace quantities. Further, with work by Ai et al. on an MoS_2_ photoelectrochemical immunosensor for detecting modifications in RNA, we realize that there could be extended use of this material in conducting biomediagnosis and epigenetics-related studies. In comparison to labor-intensive sample preparation, numerous wash steps, and long incubation times involved in enzyme-linked immunosorbent assays (ELISAs), MoS_2_ biosensors enable label-free, real-time detection with minimal sample preparation. This makes MoS_2_ a point-of-care and field-deployable food safety system game-changer. In seafood industries, for instance, Sharma et al. demonstrated the ability of MoS_2_ biosensors to identify spoilage indicators such as Xn in fish meat, which is important for preserving food freshness and avoiding contamination due to bacteria. Such sensors are particularly well-suited for on-site testing due to miniaturization and disposability in supply chains, supermarkets, and restaurants.

### 3.4. Wearable Health Devices

When it comes to electronic wearable devices, MoS_2_ proves to be a fit material because of its atomically thin structure and flexibility, which make it suitable for health monitoring and motion detection technologies as shown in Table 4. Mondal et al. aimed to develop a highly sensitive MoS_2_-based wearable sensor based on honeycomb-like MoS_2_ nanotubes to detect human skin moisture. The fabricated sensor proved to be a promising device for rapid and highly efficient response, and hence use for various healthcare and fitness monitoring applications [104]. Another study on the development of advanced wearable devices was conducted by Shi et al., aiming to develop multifunctional devices for diagnosing oral diseases like dental or healthcare applications. The gas sensor was fabricated by performing the sonication for 21 h for the mixture of the bulk-layered MoS_2_ with ethanol and deionized water, which was later filtered and dried in a vacuum oven. The powder of MoS_2_ was mixed with sulfuric acid followed by sonication to remove large flakes. Both the mixtures were mixed up using electrostatic adsorption for 1.5 h and the prepared sensor was integrated into the PET substrate. The sensor tends to exhibit exceptional linearity and effective monitoring even during facial and oral movements like swallowing and chewing [105]. A similar thermoelectric wearable device was fabricated by Guo et al. based on a gold-decorated MoS_2_ nanosheet. The main aim of the study was to develop a device that is capable of transforming the heat from the human body to wearable electronic devices. The device was fabricated by chemically exfoliating MoS_2_ and forming a layer of gold nanoparticles. MoS_2_ was exfoliated using a lithium intercalation process followed by the growth of gold particles. This prepared structure was assembled into thin films and formed into a wristband for testing. The device tends to exhibit strong mechanical stability by maintaining 97% thermoelectric performance, offering a reliable power source for electronic wearable devices [106]. Rana et al. explored the piezoresistive properties of MoS_2_ thin films for the development of a highly sensitive wearable strain sensor to monitor real-time body movements like gestures. The hydrothermal method was used to synthesize MoS_2_ and create thin films using liquid-phase exfoliation. It was developed on the flexible polyethylene terephthalate (PET) substrate and tested by changing the current during bending and straightening cycles, which were consistent and showed a balance between sensitivity and durability [107]. Xu et al. developed a large programmable film of MoS_2_ using direct laser writing for monitoring health-related signals. The sensor was fabricated by synthesizing MoS_2_ films on silicon or silicon dioxide wafers and shifting them onto a PET substrate with Au-interdigitated electrodes by creating customizable patterns in a programmable manner. The fabricated sensor exhibited excellent piezoresistive behavior, high performance, and excellent stability and was able to monitor various health-related problems. The study demonstrated the use of direct laser writing to produce high-quality MoS_2_ films, making way for advancements in healthcare technologies [108].

Vaghasiya et al. introduced wearable sensors for telehealth by using MoS_2_-based nanomaterials for health monitoring and detecting physiological markers like temperature, respiration rate, etc. The paper mainly focused on the development of more efficient, rapid-response sensors that are capable of tracking health data [109]. The key challenge in the study was to produce 2D materials on a large scale, maintaining the stability, comfort, and privacy of the data collected by the wearable electronic sensors. Five major types of wearable sensors were highlighted in this study, including pressure, strain, temperature, electrochemical, and optoelectronic. They were found to be effective for tracking different health-related parameters effortlessly and wirelessly. The fabricated sensors were sensitive enough for detecting various physiological changes, which made them suitable for personalized healthcare, and it was also concluded that personalized healthcare could be revolutionized by combining wearable devices with advanced AI/ML tools. Another study on the fabrication of wearable devices was conducted by Pang et al. using two-dimensional materials to detect human physiological signals. The use of MoS_2_ ensures the conforming of the wearable device with the skin’s surface, allowing non-invasive monitoring of important signs without any discomfort [110]. Chemical signals are detected from blood fluids like sweat and saliva with the help of MoS_2_ in wearable devices. The sensor is combined with a suitable substrate and other two-dimensional materials for improving the functionality and hence enhancing the performance, making it suitable for health monitoring systems. Moreover, it plays an essential role in wearable electronic systems because of its unique properties. Kim et al. developed a highly sensitive and flexible strain-pressure sensor with a cracked paddy-like MoS_2_ nanostructure. The researchers observed that the sensor’s performance was improved by increasing the MoS_2_ content and adjusting the concentration of MoS_2_ nanoflakes. It was successfully tested for monitoring real-time detection of human motions like blinking of an eye, and neck movement. Overall, the study demonstrated the effectiveness of using MoS_2_ for high-performance wearable devices [111]. Another study for the development of a high-performance pressure sensor using an integration of polydimethylsiloxane (PDMS) and 1T-phase MoS_2_ was conducted by Yang et al. to monitor human physiological signals like finger movements, speech recognition, and pulse measuring. The sensor was fabricated using a mixture of PDMS and curing agents in a 5:1 to 30:1 ratio along with NaCl salt [112]. The mixture was later poured and cured into a mold for 4 h at 80 °C. The combination of high electrical conductivity of 1T-MoS_2_ and the microstructure of a leaf vein tends to enhance the selectivity of the sensor and pressure response by varying the contact resistance between MoS_2_ and PDMS. Yu et al. demonstrated a self-powered MoS_2_-based nanogenerator to accelerate the healing of wounds in diabetes. The device tends to exhibit excellent electrical conductivity and efficient photothermal conversion, hence accelerating the process of wound healing in diabetic patients [113]. Also, while graphene has been widely explored for wearable health monitoring due to its excellent conductivity and flexibility, MoS_2_-based biosensors offer distinct advantages for next-generation devices. Unlike graphene, which lacks an inherent bandgap and often requires complex doping to achieve optimal sensing performance, MoS_2_’s natural semiconducting properties enable highly sensitive and selective detection of biomarkers at ultra-low concentration, critical for early disease diagnosis.

MoS_2_ wearable sensors offer extraordinary potential in next-generation healthcare and human monitoring device development. MoS_2_’s atomically thin nature, mechanical flexibility, tunable bandgap, and semiconducting nature make it an attractive replacement for traditional silicon and even graphene for wearable use. MoS_2_’s layered structure allows it to be in intimate contact with human skin, forming highly conformal, non-invasive sensing platforms for real-time physiological readings. Mondal et al., for example, described the application of honeycomb-structured MoS_2_ nanotubes for detecting human skin dampness, which demonstrates the material’s fast response and efficacy in fitness/disease diagnostics. Shi et al. also fabricated a gas sensor on a PET substrate from exfoliated MoS_2_ with similar detection of oral movements such as chewing and swallowing, which are pivotal in oral disease detection and dental care. These reports demonstrate MoS_2_’s high sensitivity, mechanical flexibility, and ability to incorporate wearable systems in everyday life without any annoyance or interference. MoS_2_’s applicability to different sensing mechanisms including piezoresistive, thermoelectric, optical, and electrochemical mechanisms tremendously enhances its scope of application in wearable health monitors. In addition, wearable sensors made from MoS_2_ nanomaterials have been applied to telemedicine and remote health monitors. Five categories of sensors were classified by Vaghasiya et al., namely pressure, strain, temperature, electrochemical, and optoelectronic, where MoS_2_’s high sensitivity allowed for detecting infinitesimal physiological changes. This not only allows for health tracking in real time but also for personalization and the addition of AI to health systems. MoS_2_’s compatibility with biofluids like sweat and saliva, demonstrated by Pang et al., supports their use in biomarker monitoring with non-invasive methods. The compatibility of substrates that mimic skin with the sensor supports wearability over long periods of time along with comfort levels for users. Kim et al. extended this with cracked paddy-like MoS_2_ nanostructures for motion sensing in real time, e.g., eye blink rates and neck movement—functionality imperative for human–computer interaction and assistive technology. Further, research by Yang et al. with MoS_2_-PDMS composites exhibited an improved sensitivity to pressures due to leaf vein-like microstructures, pointing to the use of MoS_2_ with suitable polymers in tactile sensing.

### 3.5. Other Applications

**Figure 6:**
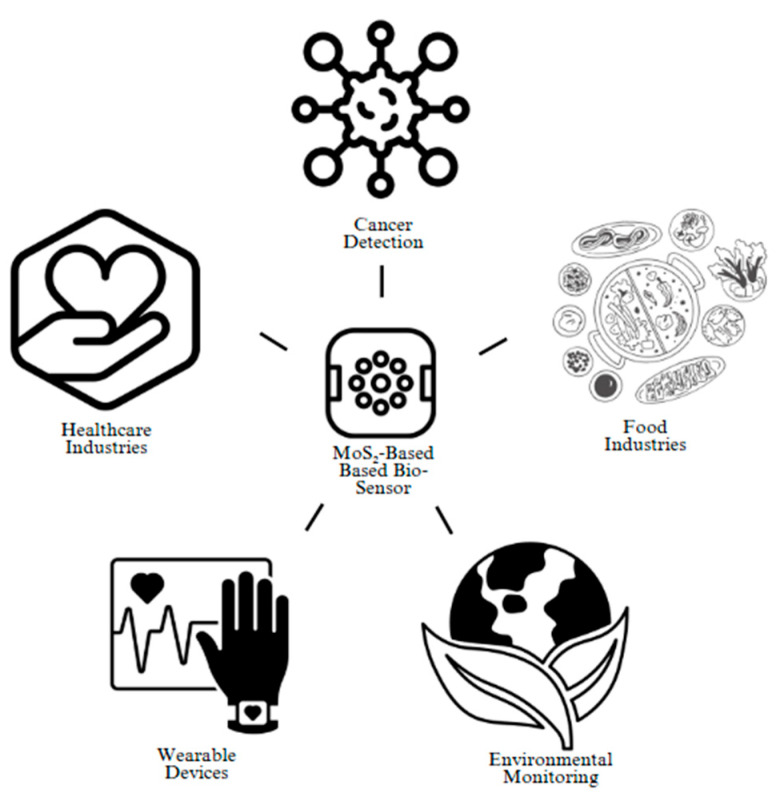
Applications of MoS_2_-based sensors in various fields.

MoS_2_ is widely integrated in the environmental industry for the identification of contaminants like inorganic compounds and poisonous gases in the environment, as shown in Table 5 and Figure 6 [114]. Guo et al. developed a sensor using MoS_2_ base along with boronic acid for the sensitive and selective detection of mercury ions in environmental water. The proposed method exhibited excellent optical properties, high quantum yield, strong fluorescence, and highly selective analysis of Hg ions in the contaminated water with a much faster response. This paper also presents an overview of a gas-phase reaction involving methane and a metal catalyst used in CVD. When methane gas splits into carbon (C) and hydrogen gas (H_2_) under specific conditions, solid carbon is deposited on a substrate as part of the fabrication process. Particularly in the production of thin films, semiconductors, and nanomaterials, this method is essential to materials science [115]. Sahatiya et al. proposed a novel approach for wireless monitoring of the environment using a cellulose paper-based MoS_2_ sensor with the help of the hydrothermal method. The fabrication of the sensor involves three steps. The initial step is the growth of MoS_2_ on the cellulose paper, which was further dipped in a seed solution followed by drying of the paper substrate at 80 °C. The second step includes the growth of Cu_2_S by using MoS_2_ on cellulose paper as a substrate. The last step is to cut the obtained Cu_2_S-MoS_2_ cellulose paper into desired pieces followed by a final finishing using silver paste. The fabricated sensor is utilized once and then disposed of and is not further used for other sensing applications. The sensor is capable of measuring the temperature, humidity, human breath, and ethanol adulteration [116]. Su et al. discussed the recent developments of two-dimensional nanomaterials for developing electrochemical sensors to detect pollutants like bacteria, pesticides, and insecticides [117]. The recent advancements and electronic properties of MoS_2_ such as direct bandgap are discussed in the study. The potential of developing fast, low-cost, and sensitive sensing devices for addressing the need for efficient monitoring tools to control the increase in environmental pollution has been discussed in this study. Singh et al. developed an MoS_2_-based nanocomposite biosensor for the selective detection of NH_3_. It was noticed that there is a high need for devices that are capable of operating under extreme conditions and the selective detection of NH_3_ becomes difficult because the latter is a mixture of various gases and humidity. Thus, there is a need to develop such MoS_2_ composite-based NH_3_ sensors to operate under such humidified environmental conditions [118]. The research mainly focused on addressing the problems related to the development of sensors, the successful synthesis of MoS_2_, and the electrical and gas sensing properties of the devices made from MoS_2_.

Chen et al. focused on developing two-dimensional TMDCs-based biosensors for environmental applications. The water quality was analyzed using a TMDC biosensor which mainly focused on detecting ions and non-ionic molecules and hence offering real-time monitoring, which is very useful for environmental analysis. However, the performance depends on the intrinsic properties. Their study highlighted the unique physical and chemical properties of TMDC [119]. It was also observed that 2D TMDCs are costly, have poor uniformity, and face major challenges with large-scale production in TMDC device fabrication. Another study for colorimetric determination of Hg ions with high sensitivity and high selectivity in environmental water was introduced by Ma et al. with the help of MoS_2_-based composites. The MoS_2_ nanosheets were synthesized and the resulting MoS_2_ nanocomposites were characterized using techniques like SEM and TEM to confirm the chemical composition. Researchers found that this method for colorimetric detection proves to be an effective and practical method for detecting the trace amounts of mercury ions in environmental water samples [120]. Bolukbasi et al. presented a precise and selective approach for electrochemical sensors based on MoS_2_ nanocomposites for paraoxon detection in tap water samples, which is a toxic organophosphorus pesticide, using the hydrothermal method. The sensor used molecular imprinting technology, leading to a highly sensitive and selective approach with a detection limit of $2.0\times 10^{-12}$ M. It was also recorded that the MoS_2_-based nanocomposites can be further utilized to determine the environmental pollutants, which also improves the voltammetric to determine the complex samples [121]. Another study was conducted by Wang et al., enhancing an MoS_2_-based electrochemical biosensor to detect bisphenol A in environmental water. The sensor was fabricated using Au nanoparticles, MoS_2_ nanoflowers, and ionic graphene for the formation of a nanocomposite. The fabricated sensor was later characterized using techniques like TEM, SEM, and X-ray diffraction, which also confirmed that the sensor tends to have a large surface area and good dispersion. The cost-effective sensor was able to hold potential for a wide range of applications in pollutant detection because of its excellent performance characteristics [122]. Amalraj et al. developed a biosensor using CuO@PDA-MoS_2_ nanospheres combined with an ssDNA probe for the detection of mercury ions and chloramphenicol (CAP) in water. For the detection process, the mercury ions played an essential role in cleaving the PS-RNA linkage in ssDNA, which releases the fluorophore, whereas the CAP interacts with the leftover ssDNA to form a complex which is released from CuO@PDA-MoS_2_. The fabricated sensors tend to high selectivity when used for real water samples, and a wide linear detection range, making it fit for environmental samples [123]. Sphere-like MoS_2_ nanosheets were used by Nehru et al., accurately monitoring the toxic pollutants like nitrobenzene in the samples of environmental water. The sensor was fabricated using the hydrothermal method by imposing MoS_2_ nanosheets on reduced graphene oxide and further characterizing using techniques like TEM, XPS (X-ray photoelectron spectroscopy), and FTIR (Fourier-transform infrared spectroscopy) [124]. It was also tested in real water samples from tap water that showed effective detection of NB with satisfactory recovery rates. Also, MoS_2_ biosensors demonstrate superior performance compared to conventional carbon nanotube (CNT)-based sensors for environmental monitoring applications. While CNT sensors have been extensively used for detecting pollutants like heavy metals and toxic gases, MoS_2_ offers enhanced selectivity through its tunable electronic properties and surface chemistry, enabling more accurate discrimination between similar contaminants. The atomically thin structure of MoS_2_ provides exceptional sensitivity, capable of detecting trace-level pollutants at concentrations below what CNT-based systems can reliably measure. Furthermore, MoS_2_ exhibits better environmental stability, particularly in aqueous conditions where CNTs are prone to oxidative degradation, making MoS_2_ particularly advantageous for long-term water quality monitoring applications.

The integration of MoS_2_ in environmental sensors is a significant leap forward in creating efficient, sensitive, miniaturized sensing platforms to identify contaminants. MoS_2_’s large surface area to volume, adjustable bandgap, and efficient adsorption properties make it particularly suitable to react well with a range of environmental contaminants such as heavy metals (for instance, mercury), toxic gases such as ammonia, and organic contaminants such as bisphenol A and paraxo-n. Hydrothermal, CVD, and aqueous-based LPE methods for synthesizing MoS_2_ nanomaterials have been found to be efficient to produce sensors that are capable of detecting trace amounts of contaminants in complex environmental matrices such as drinking water and environmental wastewater. Colorimetric and electrochemical biosensors based on MoS_2_ nanocomposite matrices have been found to demonstrate high sensitivity and specificity for detecting contaminants like Hg^2+^ ions and nitrobenzene, contributing immensely to environmental health, environmental security, and regulation. Further, the better aqueous system environmental stability of MoS_2_ over a material like carbon nanotubes (CNTs), with their avidity for water that predisposes them to oxidative breakdown, presents an unheralded advantage. The ability of MoS_2_ biosensors to detect, in real time, label-free and selective conditions under humid or hostile environmental conditions makes them extremely promising candidates for water purification, air pollution management, and agricultural farm management. Further, MoS_2_’s ability to be functionalized on paper-based, lossy substrates or microfluidic substrates adds an additional layer of economical and ecological relevance, which renders them compatible with field-based deployment. Nonetheless, their integration in environmental sensing is not without limitations. The reproducibility and upscalability of fabrication pose a major limitation. While hydrothermal and LPE fabrication methods are economical and low-tech, these methods tend to induce layer thickness, surface defects, and morphological inconsistencies that are detrimental to uniformity of performance. Conversely, technical-intensive methods like CVD, while efficient to produce high-quality, uniform-quality films, are still costly and technically demanding to use in large-scale environmental sensing systems. Another significant concern is MoS_2_-based sensors’ selectivity in complex environmental matrices. Under optimal laboratory conditions, their good sensitivity may be undermined by interference from structurally similar impurities in real-world environments or changes in environmental sample pH and ionic strength. Further, non-uniformity in synthesis, functionalization, and characterization protocols across studies renders comparison of performance levels complex and discourages commercial use. Long-term stability, especially under continuous use with exposure to changing contaminants, is also a concern. MoS_2_, being somewhat more stable than most other nanomaterials, still tends to oxidize or degrade over long timescales, especially under exposure to UV radiation or oxidizing molecules. Another shortcoming is its requirement for a complicated functionalization process that allows high selectivity. Such process steps, such as the addition of molecular imprinting or use of targeted binding probes, have a tendency to increase fabrication complexity and costs. Some MoS_2_ composites also contain environmentally unwanted chemicals or rare elements, raising questions about sustainability and ecotoxicity.

## 4. Characterization

To fully exploit the capabilities of MoS_2_ in biosensor applications, it is crucial to meticulously analyze its structural, morphological, optical, and electrical properties. Characterization approaches offer essential insights into the quality, thickness, phase composition, crystallinity, surface morphology, and chemical states of MoS_2_ nanosheets and thin films. These parameters substantially affect the efficacy and dependability of MoS_2_-based biosensors. This section provides an overview of the principal techniques utilized to evaluate MoS_2_, including Raman spectroscopy, AFM, TEM, SEM, and optical spectroscopy. Each method independently enhances the comprehension of the physical and chemical properties of MoS_2_, hence informing the optimization of production techniques and functional integration into biosensing platforms. A crucial phase in the development of efficient MoS_2_-based biosensors is the functionalization of the MoS_2_ surface or the immobilization of biomolecular recognition components, like antibodies, DNA, or aptamers. These changes facilitate selective binding to target analytes, hence improving sensor specificity and sensitivity. Prevalent functionalization techniques encompass covalent bonding, physical adsorption, and linker molecule approaches. Nonetheless, obstacles persist in attaining consistent, repeatable, and bioactive immobilization, particularly under fluctuating environmental circumstances. Additional research aimed at enhancing functionalization chemistries and immobilization techniques is essential to fully exploit MoS_2_’s capabilities in practical biosensing applications.

### 4.1. Raman Spectroscopy

Raman spectroscopy is an experimental phenomenon established by Raman and Krishnan in 1928, when they discussed the inelastic scattering of light [125,126]. The main characteristic of this technique was the presence of various radiations with varying frequencies. It is based on the principle of Raman Scattering, which is the exchange of energy between a fraction of scattered photons with that of target molecules [127]. The frequency shifts are identified by analyzing the scattered light. For irradiating the samples, it employs a radiation which is scattered from the molecule.

Lin et al. conducted research to study the thermal expansion coefficient of MoS_2_ nanosheets using temperature-dependent Raman spectroscopy, focusing on the examination of both suspended and supported MoS_2_ within a range of 77 to 557 Kelvin temperature [128]. The study mainly focused on how Raman’s modes shifted with temperature. Another study for the exploration of the dynamic interactions between the mechanical vibrations of MoS_2_ and Raman phonon modes was conducted by Yang et al., allowing direct probing of strain in the resonators of MoS_2_. The research also highlighted the influence of various factors like thermal effects and doping on the shifts observed during Raman spectroscopy, making it a dominant factor in the nonlinear regime [129]. Sam et al. proposed a method that focuses on investigating the stack configurations and interlayer interactions in MoS_2_ which affect the optoelectronic features of the material, with the help of the low-frequency Raman spectroscopy approach [130]. The study mainly focused on identifying different stacking configurations across the sample and detecting the defects and twists in the layer to update the low-frequency Raman spectra. Ghopry et al. developed a novel approach utilizing the heterostructures of intermixed WS_2_ and MoS_2_ using this spectroscopy for improving the sensitivity. The substrate was fabricated using the intermix of WS_2_ and MoS_2_, which is responsible for improving the sensitivity, rather than using the traditional methods [131]. This approach for the fabrication of surface-enhanced Raman spectroscopy (SERS) substrates using two-dimensional materials is quite cheap and scalable, offering various biosensing and environmental applications. Another study for developing a two-dimensional heterostructure of MoS_2_ along with gold particles was conducted by Alamri et al., employing a transfer-free CVD method for the growth of two-dimensional material on silicon wafers. The research demonstrated significant advancement in SERS technology, hence showing high sensitivity in comparison with that of graphene systems [132].

Raman spectroscopy does not need any sample preparation procedures and it is capable of analyzing the materials in their original state with any damage. It is capable of monitoring real-time dynamic changes that occur in the samples [133]. This process enables the detection of tiny amounts of molecules, which is required to study the low-concentration samples [134]. It is responsible for rapid data acquisition and hence reducing the time required for analysis [135]. However, Raman spectroscopy has poor sensitivity compared to the other techniques for detecting single molecules. For effective detection, it requires a large number of molecules [136].

### 4.2. Optical Spectroscopy

Optical spectroscopy refers to a process where light is used to magnify and visualize tiny objects that are not even visible to the naked eye [137]. In this process, a light source provides illumination that passes through the sample or gets reflected back. Then, the image of the sample is magnified through the lens, which stays still using a stage for fine movement during imaging.

A study by Wu et al. [138] demonstrated the use of an MoS_2_-based biosensor decorated with gold nanoparticles for the optical detection of explosives, utilizing plasmonic signal changes to achieve high sensitivity and selectivity. Kopaczek et al. proposed an approach to study the direct and indirect optical transitions in bulk and extremely thin MoS_2_ nanosheets. It was observed that while reducing the thickness of MoS_2_ from bulk to atomically thin, no spectral shifts were exhibited by the direct optical transitions whereas blue shifts were observed with indirect transitions [139]. Another study for characterizing the optical properties of monolayer and few-layered two-dimensional materials was conducted by Niu et al., aiming to investigate the dependency of thickness on the optical transitions and excitonic features of these materials. The optical spectra of different 2D materials were compared and variations were observed in the excitonic peak positions because of the variations in their electronic structures, making it suitable for various applications like designing and optoelectronic industries [140]. Posadas et al. presented a study for the real-time monitoring of CVD growth of MoS_2_ on sapphire substrates with the help of optical spectroscopy. Certain factors like growth rate and material coverage were measured concerning the changes observed in the optical transmittance spectrum [141]. Zhao et al. focused on studying the electrical characterization techniques for the confirmation and verification of the quality of material and its thickness before taking any electrical measurements. In the study, optical microscopy was combined with Raman spectroscopy to guarantee the uniformity of the MoS_2_ layer throughout the entire substrate [142].

Optical spectroscopy allows the analysis of various biological samples without any damage or alteration. It enables real-time monitoring, leading to the overall understanding of the biological structures [143]. This method is capable of operating in environments where other techniques are unsuitable because of the vacuum requirements and has compatibility with solvents, reactants, and other chemicals [144]. The use of optical microscopy enables the examination of various properties of materials like doping levels beyond the visible spectrum. It allows the ability to process and analyze the images with high accuracy [145,146]. However, optical microscopy does not go well with the samples that spread the light in various directions, which makes the images blur due to weak and unwanted light signals [147].

### 4.3. Scanning Electron Microscopy (SEM)

SEM is a technique that uses a beam of light which is focused to scan the surface of a sample [148]. During this technique, a beam of electrons is scanned over the surface of a specimen. The information about the specimen surface lies in the signals generated by the interactions between the electron beam and the atoms at the surface [149]. This method is widely used for biological and material science applications.

A study conducted by Yang et al. concentrated on the advancement and demonstration of a clean, quick, and efficient polymer-free transfer method for shifting the layers of MoS_2_ onto the TEM grids for liquid cell electron microscopy (LCEM) [150]. In the study, the SEM technique was used to capture the images where MoS_2_ layers were transferred onto TEM grids, and was also used to compare the polymer-free transfer method with that of traditional methods, revealing the advantages of the polymer-free method in reducing the damage and confirming high transfer yield. Xuan et al. presented a study to examine the impact of reaction temperature and reaction time on the morphology and crystallization of MoS_2_ which is synthesized using a hydrothermal method [151]. It was noticed that at low temperatures, MoS_2_ tends to appear as aggregated particles whereas at high temperatures, it tends to appear as nanosheets with more distinctive layers. Another study to examine the effect of nickel on MoS_2_ growth was conducted by Kondekar et al., seeking the morphology and the size of the grain of the MoS_2_ films [152]. The surface morphology of the MoS_2_ films was observed using the SEM images, which were also used to assess the grain size of MoS_2_. Tyagi et al. aimed to analyze the morphology, structure, and thickness of the layer of MoS_2_ films using SEM. SEM images provided a detailed view of films, ensuring the vertical alignment of the MoS_2_ layers, large area, and uniform growth of the film [153]. Another similar study was conducted by Hossain et al., aiming to investigate three important factors, namely the thickness, uniformity, and morphology of MoS_2_. All three essential factors were analyzed using SEM images [154].

The primary benefit of SEM is its capability to use SEM detectors [155]. It provides high-resolution images which makes it effective in examining the detailed morphology of different structures [156]. However, SEM cannot analyze the chemical composition of various samples unless it is coupled with other techniques, and also the sample prepared through SEM cannot be used further for analysis purposes [157].

### 4.4. Transmission Electron Microscopy (TEM)

TEM provides images with high resolution to understand the internal structure of the small samples. It is a process in which high-energy electrons are generated, accelerated, and focused into a thin beam with the help of electromagnetic lenses [158]. The beam is passed through an extremely thin specimen where it has to interact with the sample. After passing through the sample, the collected electrons form an image on a screen which can be magnified or analyzed at the atomic resolution. Based on the angle and type of collected electrons, various modes like bright-field, dark-field, and diffraction are allowed using TEM [159].

Zhang et al. focused on investigating the behavior of MoS_2_ under ultrafast photoexcitation with the help of TEM. The researchers combined traditional electron microscopy with ultrafast laser pulses, which allowed them to observe the initiation and propagation of strain waves directly [160]. The data provided by TEM also monitor the defects such as step edges, influencing the dynamics of the material. Convergent beam electron diffraction (CBED) and TEM were used by Hovden et al. to ascertain the thickness and stacking order of 2D materials [161]. To comprehend the structural characteristics of the 2D materials, the study gave accurate measurements of the layer thickness. Wen et al. suggested a technique for accurately analyzing monolayer materials such as MoS_2_ to explore both light and heavy atoms [162]. This cutting-edge imaging technology results in the creation of an effective TEM tool for the next research and applications. Another study conducted by Zhang et al. aimed to develop a system to reveal the dislocations that might occur at the MoS_2_ interface. Two types of dislocations were observed: Pure Misfit (orientation is parallel to the interface) and Angled Dislocations (dislocation at 60 degrees with the interface) [163]. Wang et al. focused on the development of a top-down method for enhancing the catalytic activity of MoS_2_ for H_2_ evolution reactions. TEM was used in the study to visualize the etching process and form active edge sites for the real-time monitoring of the steam etching of MoS_2_ [164].

TEM has both spatial and high temporal resolution and it also provides the direct visualization of any kind of changes seen in either structural or morphologic distributions [165]. It has the advantage of high resolution, which makes it suitable for in-depth investigation of samples like in Earth science [166]. TEM was crucial for confirming the structural and epitaxial relationships between different materials [167]. This type of microscopy is also used for detecting defects such as dislocations and vacancies, which may affect the properties of two-dimensional materials. However, it is difficult to completely ignore the electron-induced damage that occurred during TEM measurements [168].

### 4.5. Atomic Force Microscopy (AFM)

AFM was first introduced in 1986 and has a wide range of applications in biology, chemistry, nanoscience, and the healthcare industry [169]. The surface of a sample is scanned with the help of a sharp probe attached to a flexible cantilever using AFM. During this technique, the end which scans the sample is kept near to the sample, and the forces between them cause the cantilever to deflect, which is later measured by a laser light onto a photodetector [170]. It senses various forces like mechanical, adhesion, and electrostatic in different modes like contact, non-contact, and tapping. These forces provide information about mechanical properties like stiffness, adhesion, and elasticity by recording the force–distance curves.

Acikgoz et al. conducted a study where AFM was used to measure the frictional forces on monolayer and bulk MoS_2_. 3D images of MoS_2_ were taken using AFM to distinguish the single-layered regions from the bulk regions. It allowed the researchers to explore the variations that occurred in frictional forces with the sliding speed [171]. Giannazzo et al. proposed a study where AFM was used in two forms (TAFM—Tapping mode Atomic Force Microscopy and CAFM—Conductive mode Atomic Force Microscopy) for characterizing single-layered MoS_2_ grown using CVD. TAFM was used to measure the thickness for analyzing the domain structure and boundaries whereas CAFM was used to quantify the local electrical conductivity of MoS_2_ domains [172]. Wu et al. employed AFM for the mechanical and tribological analysis of two-dimensional materials. The primary goal of AFM is to assess mechanical properties like strength and elasticity, whereas the other mode of AFM is used to quantify these properties by determining the normal forces between the sample’s surface and probe tip [173]. Another study was conducted by Lipatov et al. which focused on characterizing the electromechanical properties of the 2D $MoS_{2}$ materials. In AFM, the MoS_2_ surface is scanned by applying a small voltage to the tip which generates an electromechanical deformation with the surface. The study showed that by using various modes of AFM, the ferroelectric properties of the MoS_2_ surface can be explored [174]. Liu et al. used AFM to study the interlayer friction and superlubricity between the two-dimensional materials. The interlayer friction was directly measured by wrapping the 2D flakes on AFM tips, which allowed the researchers to examine the friction between these materials [175]. The frictional properties or the formation of atomic resolution were explored using these AFM tips at the nanoscale level.

AFM is known to achieve high spatial resolution, which makes it suitable for studying different atomic friction patterns and providing detailed information about the force maps. This approach is capable of measuring the nanoscale frictional forces that occur between the AFM tips and the substrate [176]. This technique is also compatible with atomic manipulation, making it feasible for inducing chemical reactions with the end tip of the microscope [177]. However, AFM requires the adsorption of nanoparticles onto the support surfaces like mica and silicon wafers.

## 5. Future Perspectives and Conclusions

The future application as a multifunctional 2D material is promising for an extremely diverse range of industrial and scientific applications such as MoS_2_ and graphene [178,179]. With continuous exploration of its unique structural, electronic, optical, and mechanical properties, MoS_2_ is all set to be a keystone for future technological developments in medicine, environmental sensing, energy harvesting, bendable electronics, and so on. One of the most intriguing aspects of MoS_2_ is that it is an intrinsic semiconducting material, which represents a significant advantage over graphene for electronic- and sensing-related applications that require an intrinsic bandgap. Its high surface area to volume, mechanical flexibility, and biocompatibility make it an exceptional material for designing sensitive, miniaturized, and energy-efficient sensors for real-time sensing and diagnosis. In biosensing, MoS_2_ is poised to revolutionize disease detection and environmental monitoring owing to its exceptional electrochemical and optical sensitivity. Future directions will be to hybridize MoS_2_ with other nanomaterials such as noble metals, polymers, and carbon nanotubes in order to enhance selectivity, amplify the signal, and make it long-lasting. Future applications in emerging research will be integrating MoS_2_ with lab-on-a-chip instruments and point-of-care kits, which will become inevitable for early disease identification, epidemic containment, and personalized medicine. Combing MoS_2_-based sensors with microfluidics and smartphone integration also promises to lead to fully automated, portable diagnoses with real-time tracking capabilities, even in resource-limited environments and telemedicine systems. While chemical vapor deposition (CVD), hydrothermal growth, and liquid-phase exfoliation have promisingly started to show potential, wafer-scale, defect-free, layer-controlled MoS_2_ growth remains a major hurdle. Investigation of scalable, clean synthesis methods with well-controlled shape and phase purity will be important. Further, 1T, 2H, and mixed-phase MoS_2_ synthesis exploration, together with heterostructure design, could lead to new functionalities that are application-specific. The long-term biocompatibility and environmental friendliness of MoS_2_-based nanomaterials will need to be exhaustively tested to enable safe and benign deployment, particularly in medicine and consumer products. In conclusion, the exploration of MoS_2_ material as biosensors led to significant advancement in various fields like medicine, environmental monitoring, and the food industry. Its excellent electronic properties like high surface-to-volume ratio, high surface area, tunable bandgap, and strong biocompatibility to detect various biomolecules make it an ideal material for biosensing applications with high sensitivity and accuracy. The layered structure of the material allows effective interaction, making it effective to detect various biomarkers. Despite these promising attributes, it faces several technical challenges including the optimization of the fabrication process to improve the performance and enhance the reproducibility. Challenges such as large-scale production, enhancing specificity towards the target analytes, and achieving low-cost production and long-term stability persist for future research. Future developments should focus on the advancement of MoS_2_-based biosensors for improving the accuracy and expanding the functionality. Overcoming these challenges will lead to the development of a robust, cost-effective, and scalable system for biosensing applications.

## Figures and Tables

**Table 1:** Studies of cancer-based applications.

S. No	Author Name	Publication Year	Findings/Results
1.	[73]	2022	Investigated a five-layered surface plasmon resonance (SPR) biosensor using $MoS_{2}$, which is capable of detecting different types of cancerous cells, such as breast cancer and blood cancer. The sensor has five layers coupling with a prism at the end which is responsible for wave vector matching, a crucial step for SPR condition to occur. The thickness of the Ag layer present in the sensor determines the performance of the designed biosensor. This $MoS_{2}$-based biosensor is typically based on Kretschmann configuration and can easily detect the changes in the concentration of biomolecules with high sensitivity, thus enabling the early-stage detection of cancer.
2.	[74]	2022	An $MoS_{2}$-based biosensor was developed to detect lung cancer cell types in hydroplegia, leading to a significant reduction in long waiting times for cancer detection in hospitals. In this study, n-type $MoS_{2}$ were grown through a CVD process and a thin film of p-type $Cu_{2}O$ was grown using electrochemical deposition. The crystal structure of the $MoS_{2}$ flakes grown is analyzed using the TEM technique. The $MoS_{2}$-grown flakes undergo preliminary processing where the sample is transferred onto a $Cu_{2}O$ thin film to complete the p-n heterogeneous structure.
3.	[75]	2020	${MoS}_{2}$-based electrochemical sensor was fabricated, which focused on the highly selective detection of GSH and the quantification of GSH concentration for investigating the perspective of biopsy-free cancer detection. The fabrication of the sensor was completed using standard semiconductor processing techniques. The Glutathione-S-Transferase (GST) was immobilized on the surface of $MoS_{2}$ and GSH was determined by the electrochemical activity of GSH and CDNB in the presence of GST. The study claimed that the $MoS_{2}$-based sensor exhibits excellent stability and repeatability, making it suitable for cancer diagnosis and quantification.
4.	[76]	2022	An $MoS_{2}$-based nanosensor was fabricated to detect Tumor Necrosis Factor (TNF) in cancer patients using the electrophoretic deposition method. TNF played an important role in apoptosis and cancer and this was the first biosensor fabricated to detect TNF in cancer patients. The study also proved that $MoS_{2}$ is a promising material for the fabrication of this biosensor because of its 3D structure and high surface area.
5.	[77]	2018	$MoS_{2}$-based fluorescence sensor was developed for the early-stage detection of tumors in women’s breasts. The fabricated biosensor tends to have high sensitivity, efficiency, fast analysis, and low cost, making it suitable for miR-21 detection in breast cancer patients. In this study, $MoS_{2}$ contacts a fluorescent dye-labeled DNA probe for fabricating a fluorescence sensor and is further followed by a DNA-miRNA hybridization process. The miR-21 is detected by monitoring the change in fluorescence signal before and after the process of DNA-miRNA hybridization.
6.	[78]	2020	Another study for the detection of cancer cells using an $MoS_{2}$ biosensor based on Chalcogenide $MoS_{2}$ Au-Ag nanocomposites was provided, which has higher sensitivity and satisfactory repeatability and stability. $MoS_{2}$ was successfully synthesized and proved to be a potential application value to improve electrochemical detection performance. The peaks of Au and Ag confirmed the successful electrodeposition of two metals on the $MoS_{2}$-modified electrode. The components of $MoS_{2}$-Au-Ag were studied and analyzed using energy dispersive spectroscopy (EDS). The results of the study claimed that the modified glass carbon electrode has high sensitivity and the structure and morphology of the fabricated sensor were investigated by TEM.
7.	[79]	2023	A biosensor was fabricated using $MoS_{2}$ for detecting and analyzing the level of cancer in lung cancer cells, where a high-temperature furnace tube system was used for the formation of $MoS_{2}$ thin films on solar silicon substrates with the help of CVD technique. It is the quickest method to provide the most accurate analytical information, where a thin layer of $MoS_{2}$ is grown on a light-absorbing layered substrate of silicon with the help of CVD method. Their results showed that the fabricated device was improved by 38% in comparison with the already existing devices when a layer of $MoS_{2}$ was added to the photoelectrochemical signal; and the larger the number of cancer cells measured, the greater the photocurrent.
8.	[80]	2024	A photoelectrochemical biosensing chip based on $MoS_{2}$ was developed to achieve better sensitivity through a straight interdigitated zigzag electrode. There are two primary procedures for the experiment to occur; generating the electron–hole pairs by the photoelectric effect is the initial step and the second step involves the occurrence of an electrochemical reaction between the electron–hole pairs and samples produced by the photoelectric effect. Research was also conducted to examine the correlation between the number of modified cells and the photocurrent.
9.	[81]	2023	$MoS_{2}$ was employed as a tunable electrochemical biosensor for the detection of cancer biomarkers. It was observed that the cancer biomarkers discovered till now tend to exist in blood, tears, urine, oral fluids, and other tissues. The feature of $MoS_{2}$ to stimulate the enzymes for the changing the substrate color is used for the detection of cancer biomarkers. The study also claimed that the process involved is low-cost, simple, and has high sensitivity and faster speed as compared to the other traditional methods used for cancer biomarker detection.
10.	[82]	2022	Tumour-targeted chemo-photodynamic combination therapy was used to develop a multifunctional nanoplatform for targeted cancer therapy. The platform developed in the study consisted of upconversion nanoparticles combined with $MoS_{2}$ quantum dots. The main aim of the study was to develop multifunctional nanoplatforms for improving the precision and efficacy in cancer treatments by minimizing the side effects.

**Table 2:** Studies of medical industry-based applications.

S. No	Author Name	Publication Year	Findings/Results
1.	[84]	2019	The study claimed that two-dimensional $MoS_{2}$ nanosheets can also be used in drug delivery vehicles because of their planar structure and high surface-area-to-volume ratio. The author highlighted the importance of CVD in the deposition of thin films with excellent uniformity, and conformality. In the study, $MoS_{2}$ nanosheets provided a versatile platform for drug delivery and the research focused more on the synthesization and functionalization of thin nanosheets to improve their drug-loading capacity, stability, and targeting efficiency. Also, nanosheets are being used for the electrochemical detection of glucose and dopamine because of their high sensitivity.
2.	[85]	2024	The study showed that the $MoS_{2}$ nanomaterials exhibit excellent absorption in the near-infrared window, making them suitable for controlled drug release, which enhances the accumulation of drugs at the tumor site by minimizing the side effects on the healthy tissues.
3.	[86]	2020	The study claimed that $MoS_{2}$-based biosensors are highly effective in photothermal therapy in which near-infrared light is converted to heat for inducing localized thermal ablation of cancer cells and tissues. These sensors are also capable of detecting biological molecules and other analytes, making them valuable for medical industries.
4.	[87]	2021	In the study, the progress that has been made in TMDCs for medical examinations is dicussed for the detection of enzymes and other biological molecules. In this method, a special CVD method is applied in the synthesization of two-dimensional TMDCs, where the precursors containing transition metal elements pass through a furnace as gas, and react at a very high temperature with the carrier gas to form a layer on the substrate.
5.	[88]	2018	The DNA of chikungunya virus was detected using thin-layered $MoS_{2}$ nanosheets based on disposable biosensors. The proposed biosensor exhibited a maximum range of 100 μM and a minimum range of 0.1 nM towards the chinkungunya virus DNA. The fabricated sensor possesses less response time, high linearity, and economic feasibility, which make it useful for miniature applications. The selectivity of the biosensor was investigated by comparing the current response obtained from PDNA/$MoS_{2}$NSs and TDNA/$MoS_{2}$NSs with non-complimentary DNA. The comparative study showed that the response of PDNA is nearly equal to that of non-complimentary DNA. Also, the selectivity of the sensor was determined by keeping the concentration of the non-complementary nucleotide sample three orders higher than that of the complementary sample.
6.	[89]	2019	A highly sensitive biosensor was developed using $MoS_{2}$ films for the detection of trisomy 21 syndrome DNA. The CVD technique was used to grow the $MoS_{2}$ film on the silicon or silicon dioxide substrate. The researchers claimed that the developed biosensor has a wide range of applications in the medical field like detecting chromosome 21 in blood samples of pregnant women, and because of their low cost and high sensitivity, they are suitable for large-scale applications in the medical industry. The fabricated sensor could detect DNA even at extremely low concentrations, with a detection limit under 100 aM, and provides a high response rate with high sensitivity and high specificity in detecting DNA sequences, making the sensor effective for Down syndrome detection.
7.	[90]	2024	An $MoS_{2}$-based biosensor was fabricated and optimized for the detection of Tau protein, which is very crucial for the early-stage detection of Alzheimer’s disease. The study aimed to provide four different biofunctionalization methods to enhance the performance of biosensors. It was recorded that the fabricated sensor exhibited high sensitivity for the detection of Tau protein. The performance of the biosensor was dependent on the four different methods of biofunctionalization, which is also responsible for enhancing the sensitivity and specificity of the fabricated biosensor.
8.	[91]	2021	A label-free electrochemical biosensor was developed to detect Escherichia coli with high sensitivity and high selectivity. The hydrothermal technique was used for the synthesization of $MoS_{2}$ by combining it with PANI using in situ oxidative polymerization and fabricating the sensor on the glassy carbon electrode. The sensor was fabricated by combining AuNPs, PANI, and $MoS_{2}$ using the green hydrothermal method, followed by situ oxidative polymerization for enhancing the electrical conductivity and surface area, and the chemical reduction method for improving the sensitivity of the biosensor. The study claimed that the sensor exhibited high sensitivity and selectivity with a low detection limit, making it a promising material for early detection of the pathogen in clinical, food, and environmental monitoring.
9.	[92]	2023	The study aimed to develop an $MoS_{2}$-based microfluid biosensor for the immune detection of Toxoplasma gondii by fusing single-mode fiber with the thin-core fiber using arc discharging and flame heating. The study focused on developing a microfluid biosensor based on $MoS_{2}$ for the detection of Toxoplasma gondii, a zoonotic parasite. The developed biosensor showed excellent properties, proving its potential applications in biomedical fields to detect Toxoplasma gondii, with a detection range from 1 pg/mL to 10 ng/mL, and a limit of detection as low as 87 fg/mL.
10.	[93]	2023	García et al. provided a study highlighting different nanomaterials and their potential applications in advance sensing technologies. The study focused on the development of water purification systems for the removal of pollutants, detection of food contaminants, and monitoring of crop health.

**Table 3:** Studies of food industry-based studies.

S. No	Author Name	Publication Year	Findings/Results
1.	[94]	2022	A study was provided on the importance of biosensors based on two-dimensional materials for food safety. It was noticed that nitrite content was rapidly increasing in crops because of an increase in inorganic fertilizers sprayed in the fields. However, the nitrite content tends to enter the human body along with the food products, which converts the hemoglobin into methemoglobin and hence disrupts the oxygen delivery system in the human body, making it crucial to detect and control the nitrite content effectively. Therefore, two-dimensional-based biosensors play a vital role in food safety and biomolecular detection.
2.	[95]	2022	A study for the detection of synthetic colors in food samples aimed to reduce the side effects of artificial colors in humans. The main goal of this study was to synthesize $MoS_{2}$-based biosensors for modifying the electrodes to detect sunset yellow FCF synthetic food dye in food samples and this $MoS_{2}$-based biosensor was successfully applied to detect FCF in real-life samples. However, it was observed that the stability of the biosensor, i.e., the modified electrode, was excellent.
3.	[96]	2023	$MoS_{2}$-based sensor was grown on $g-C_{3}N_{4}$ nanotubes to determine vanillin in food samples using the hydrothermal method. Vanillin is an artificial flavoring agent used in perfumes, food products, and beverages, and is beneficial when used in limited quantities. However, the overdose of VN is hazardous and needs to be detected using a cost-effective $g-C_{3}N_{4}$-grown nanotubes approach. The proposed method used for the detection of vanillin in food samples proves to be a very cost-effective and more attractive strategy for food additive detection.
4.	[97]	2019	A simple hydrothermal method was proposed for the preparation of $MoS_{2}$-based nanocomposite for detecting terbutaline sulfate in food samples that were characterized later using techniques like TEM and SEM. The researchers used fresh samples of pork for testing and claimed that the fabricated sensor had excellent electrolytic properties.
5.	[98]	2023	$MoS_{2}$-based user-friendly sensor was developed to detect Xanthine in fish meat. Keeping fish meat fresh and free from bacteria like Vibrio and Salmonella is a major concern as fish meat has a large market in various industries like pharmaceuticals for multiple medications or fish oil supplements. Xanthine is one such bacteria found in rotten fish and the level of Xn increases with an increase in the storage time of fish meat, making it necessary to keep track of Xn in fish meat for diagnosing and monitoring diseases.
6.	[99]	2017	Carbon nanotubes coated with $MoS_{2}$ were used for detecting chloramphenicol in food samples like honey, milk, and powdered milk. Chloramphenicol is a veterinary drug used in the treatment of infectious diseases in animals but an overdose of this drug can lead to serious chronic diseases such as aplastic anemia and bone marrow depression, which makes it important to monitor the level of CAP drugs in food items. There are various techniques used for the monitoring of CAP levels in food items but electrochemical methods are low-cost, simple, and portable as compared to other traditional analytical approaches.
7.	[100]	2020	Carbon nanotubes based on two-dimensional $MoS_{2}$ were used to detect Thiabendazole (TBZ) residues in food samples. The sensor is fabricated using multiwall carbon nanotubes aligned with layered $MoS_{2}$ nanosheets, enhancing the electrocatalytic properties of multiwall carbon nanotubes with $MoS_{2}$. The fabricated sensor exhibits excellent properties for the detection of TBZ in food samples with high accuracy and safe testing.
8.	[101]	2022	$MoS_{2}$-based electrochemiluminescence (ECL) biosensors were used for ensuring food safety by detecting contaminants like pesticides and food pathogens in various food samples. There are different types of ECL biosensors available for the food sensing applications and all are well known for their high sensitivity, selectivity, and efficiency, especially for examining miniature devices. This approach for sensing the food samples is cost-effective and biocompatible.
9.	[102]	2022	The study focused on developing biosensor technology mainly in the food and healthcare industry. The study used novel nanomaterials which improve the sensitivity, selectivity, and stability of the biosensors.
10.	[103]	2021	A photoelectrochemical immunosensor based on $MoS_{2}$ nanocomposites was developed, aiming to detect N6-methyladenosine, an essential RNA modification. The biosensor was fabricated by assembling $MoS_{2}$ heterojunction, gold nanoparticles, and an m6A antibody. The main aim of the study was to improve the sensitivity and selectivity of the biosensor, hence making it suitable for various environmental and biological applications.

**Table 4:** Studies of wearable health device applications.

S. No	Author Name	Publication Year	Findings/Results
1.	[104]	2020	A highly sensitive $MoS_{2}$-based wearable sensor based on honeycomb-like $MoS_{2}$ nanotubes was developed to detect human skin moisture. The sensor was fabricated using $MoS_{2}$ nanotubes based on anodic aluminum oxide (AAO)-assisted MoS_2_ honeycomb structure (AMHS) with the help of a vacuum filtration (VM) process for the detection of human skin humidity under different physical conditions and regional sweat rates. The fabricated sensor proved to be a promising device for rapid and highly efficient response, and hence use for various healthcare and fitness monitoring applications.
2.	[105]	2023	A study on the development of advanced wearable devices was conducted to develop multifunctional devices for diagnosing oral diseases like dental or healthcare applications. The gas sensor was fabricated by performing the sonication for 21 h for the mixture of the bulk-layered $MoS_{2}$ with ethanol and deionized water, which was later filtered and dried in a vacuum oven. The sensor tends to exhibit exceptional linearity and effective monitoring even during facial and oral movements like swallowing and chewing.
3.	[106]	2018	A similar thermoelectric wearable device was fabricated based on gold-decorated $MoS_{2}$ nanosheet. The main aim of the study was to develop a device which is capable of transforming the heat from human body to wearable electronic devices. The device was fabricated by chemically exfoliating thin nanosheets of $MoS_{2}$ and forming a layer of gold nanoparticles to enhance its thermoelectric properties. $MoS_{2}$ was exfoliated using a lithium intercalation process followed by the growth of gold particles. This prepared structure was assembled into thin films and formed into a wristband for testing. The device tends to exhibit strong mechanical stability by maintaining 97% thermoelectric performance, offering a reliable power source for electronic wearable devices.
4.	[107]	2020	Hydrothermal method was used to synthesize $MoS_{2}$ and create thin films using liquid-phase exfoliation. The sensor was fabricated on the flexible polyethylene terephthalate (PET) substrate and tested by changing the current during bending and straightening cycles, which were consistent and showed a balance between sensitivity and durability.
5.	[108]	2021	A large programmable film of $MoS_{2}$ was developed using direct laser writing for monitoring health-related signals. The sensor was fabricated by synthesizing $MoS_{2}$ films on silicon or silicon dioxide wafers and shifting it onto a PET substrate with Au interdigitated electrodes by creating customizable patterns in a programmable manner. The fabricated sensor exhibited excellent piezoresistive behavior, high performance, and excellent stability. The sensor was able to monitor various health-related problems like respiratory rate, vocal cord vibrations, and radial artery pressure. The study demonstrated the use of direct laser writing to produce high-quality $MoS_{2}$ films, making way for advancements in healthcare technologies.
6.	[109]	2023	Wearable sensors were introduced for telehealth by using $MoS_{2}$-based nanomaterials for health monitoring and detecting physiological markers like temperature, respiration rate, etc. The paper mainly focused on the development of more efficient, rapid-response sensors that are capable of tracking health data. The key challenge in the study was to produce 2D materials on a large scale, maintaining the stability, comfort, and privacy of the data collected by the wearable electronic sensors. Five major types of wearable sensors were highlighted in the study, including pressure, strain, temperature, electrochemical, and optoelectronic. They were found to be effective for tracking different health-related parameters effortlessly and wirelessly. The fabricated sensors are sensitive enough for detecting various physiological changes, which makes them suitable for personalized healthcare, and it was also concluded that personalized healthcare could be revolutionized by combining wearable devices with advanced AI/ML tools.
7.	[110]	2020	Wearable devices were fabricated using two-dimensional materials to detect human physiological signals. The use of $MoS_{2}$ ensures the conforming of the wearable device with the skin’s surface, allowing non-invasive monitoring of important signs without any discomfort. Chemical signals are detected from blood fluids like sweat and saliva with the help of $MoS_{2}$ in wearable devices. The sensor is basically combined with a suitable substrate and other two-dimensional materials for improving the functionality and hence enhancing the performance, making it suitable for health monitoring systems. Moreover, $MoS_{2}$ is known to play a vital role in wearable electronic systems because of its unique properties.
8.	[111]	2018	A highly sensitive and flexible strain-pressure sensor was fabricated with a cracked paddy-like $MoS_{2}$ nanostructure. The researchers observed that the sensor’s performance was improved by increasing the $MoS_{2}$ content and adjusting the concentration of $MoS_{2}$ nanoflakes. The fabricated sensor was successfully tested for monitoring real-time detection of human motions like blinking of an eye, and neck movement. Overall, the study demonstrated the effectiveness of using $MoS_{2}$ for high-performance wearable devices.
9.	[112]	2020	A high-performance pressure sensor was developed using an integration of polydimethylsiloxane (PDMS) and 1T-phase $MoS_{2}$ to monitor human physiological signals like finger movements, speech recognition, and pulse measuring. The sensor was fabricated using a mixture of PDMS and curing agents in 5:1 to 30:1 ratio along with NaCl salt. The mixture was later poured and cured into a mold for 4 h at 80 °C. The combination of high electrical conductivity of 1T-$MoS_{2}$ and microstructure of a leaf vein tends to enhance the selectivity of the sensor and pressure response by varying the contact resistance between $MoS_{2}andPDMS$.
10.	[113]	2024	A self-powered $MoS_{2}$-based nanogenerator was developed to accelerate the healing of wounds in diabetes. The device tends to exhibit excellent electrical conductivity and efficient photothermal conversion, hence accelerating the process of wound healing in diabetic patients.

**Table 5:** Studies on environment-based applications.

S. No	Author Name	Publication Year	Findings/Results
1.	[115]	2020	A fluorescent sensor was developed using $MoS_{2}$ along with boronic acid for the sensitive and selective detection of mercury ions in environmental water. The proposed method exhibited excellent optical properties, high quantum yield, strong fluorescence, and highly selective analysis of mercury ions in the contaminated water with a much faster response.
2.	[116]	2018	A novel approach was proposed for wireless monitoring of the environment using a cellulose paper-based $MoS_{2}$ sensor with the help of the hydrothermal method. The fabricated sensor is utilized once and then disposed off and is not further used for other sensing applications, which increases the accuracy of the sensing data. The sensor is capable of measuring the temperature, humidity, human breath, and ethanol adulteration.
3.	[117]	2018	The recent advancements and electronic properties of $MoS_{2}$ such as direct bandgap are discussed in the study. The potential of developing fast, low-cost, and sensitive sensing devices for addressing the need for efficient monitoring tools to control the increase in environmental pollution is discussed in the study.
4.	[118]	2021	An $MoS_{2}$-based nanocomposite biosensor for the selective detection of ${\mathrm{NH}}_{3}$ was developed. It was noticed that there is a high need for devices that are capable of operating under extreme conditions and the selective detection of $NH_{3}$ becomes difficult because the latter is a mixture of various gases and humidity. Thus, there is a need to develop such $MoS_{2}$ composite-based $NH_{3}$ sensors to operate under such humidified environmental conditions. The research mainly focuses on addressing the problems related to the development of sensors, the successful synthesis of $MoS_{2}$, and the electrical and gas sensing properties of the devices made from $MoS_{2}$.
5.	[119]	2020	The water quality was analyzed using a TMDC biosensor which mainly focused on detecting ions and non-ionic molecules, hence offering real-time monitoring, which is very useful for environmental analysis. However, the performance depends on the intrinsic properties. Their study highlighted the unique physical and chemical properties of TMDC which make it suitable for the fabrication of FET biosensors. It was also observed that 2D TMDCs are costly, have poor uniformity, and face major challenges with large-scale production in TMDC device fabrication.
6.	[120]	2019	Colorimetric determination of mercury ions with high sensitivity and high selectivity in environmental water was introduced with the help of $MoS_{2}$-based composites. The $MoS_{2}$ nanosheets were synthesized and the resulting $MoS_{2}$ nanocomposites were characterized using techniques like SEM and TEM to confirm the chemical composition. The research aimed to synthesize with uniform AuNPs on the $MoS_{2}$ nanosheets and investigate the peroxidase-like properties for the quantitative analysis of mercury ions with high sensitivity and selectivity. Researchers found that this method for colorimetric detection proves to be an effective and practical method for detecting the trace amounts of mercury ions in environmental water samples.
7.	[121]	2022	A precise and selective approach was presented for electrochemical sensors based on $MoS_{2}$ nanocomposites for paraoxon detection in tap water samples, which is a toxic organophosphorus pesticide, using the hydrothermal method. The sensor used molecular imprinting technology, leading to a highly sensitive and selective approach with a detection limit of $2.0\times 10^{-12}\;M.$ It was also recorded that the $MoS_{2}$-based nanocomposites can be further utilized to determine the environmental pollutants, which also improves the voltammetric to determine the complex samples.
8.	[122]	2021	An $MoS_{2}$-based electrochemical biosensor was used to detect bisphenol A in environmental water. The sensor was fabricated using Au nanoparticles, $MoS_{2}$ nanoflowers, and ionic graphene for the formation of a nanocomposite. The cost-effective sensor was able to hold potential for a wide range of applications in pollutant detection because of its excellent performance characteristics.
9.	[123]	2022	A biosensor was developed using CuO@PDA-$MoS_{2}$ nanospheres combined with an ssDNA probe for the detection of mercury ions and chloramphenicol (CAP) in water. For the detection process, the mercury ions played an essential role in cleaving the PS-RNA linkage in ssDNA, which releases the fluorophore, whereas the CAP interacts with the left over ssDNA to form a complex which is released from CuO@PDA-$MoS_{2}$. The fabricated sensors tend to have high sensitivity with a detection limit of 86 pM for mercury ions and 45 pM for CAP, high selectivity when used for real water samples, and a wide linear detection range, making them fit for environmental samples.
10.	[124]	2022	Sphere-like $MoS_{2}$ nanosheets were used to accurately monitor the toxic pollutants like nitrobenzene in the samples of environmental water. The sensor was fabricated using the hydrothermal method by imposing $MoS_{2}$ nanosheets on reduced graphene oxide and further characterizing using techniques like TEM, XPS (X-ray photoelectron spectroscopy), and FTIR (Fourier-transform infrared spectroscopy). The sensor tends to have high sensitivity with a detection limit as low as 0.0072 μM, and high selectivity for NB detection. It was also tested in real water samples from tap water that showed effective detection of NB with satisfactory recovery rates.

## Data Availability

The data presented in this study are available in this article upon considerable request to the corresponding author (Hsiang-Chen Wang).

## Appendix A

**Table A1:** Studies of mechanical exfoliation.

Author Name	Paper Title	Publication Year	Findings/Results
[19]	Mechanical exfoliation of two-dimensional materials	2018	A criterion to measure the feasibility of peeling off 2D sheets from the substrate without causing any fractures was proposed, which aimed to optimize the peeling process for high efficiency and better yield. The theoretical models were validated by comparing them using the coarse-grained molecular dynamics (CGMDs) simulation results. The results concluded that peeling angle and adhesive strength are the two most important factors for controlling the peeling process.
[20]	Universal mechanical exfoliation of large-area 2D crystals	2020	A method was developed for assisting the exfoliation of monolayered $MoS_{2}$ from their bulk crystals, and it was mainly effective where exfoliation is difficult using conventional methods.
[21]	Ultrasensitive WSe_2_ field-effect transistor-based biosensor for label-free detection of cancer in point-of-care applications	2021	A method to develop label-free detection of cancer in point-of-care applications using mechanical exfoliation methods. Bulk crystals of $WSe_{2}$, which is a layered material, are taken, which also makes it possible to separate these layers mechanically, which are later transferred to silicon or silicon dioxide substrates. Techniques such as Atomic Force Microscopy (AFM) were used to ensure the quality and thickness of the exfoliated $WSe_{2}$ layers.
[22]	High-Throughput Mechanical Exfoliation for Low-Cost Production of van der Waals Nanosheets	2023	An efficient method for developing thin nanosheets of $MoS_{2}$, using a roll-to-roll setup approach that creates excellent flake density, was developed. This nanosheet production method presents a promising solution for the cost-effective production of ultrathin sheets while maintaining good electronic and optoelectronic performance.
[23]	Surface characterization of MoS_2_ atomic layers mechanically exfoliated on a si substrate	2020	Scotch tape method was proposed to mechanically exfoliate a thin layer of $MoS_{2}$ flakes from bulk $MoS_{2}$ crystals and further transfer the flakes on a polished and undoped Si substrate. In the study, relative EPES measurements were used for evaluating IMFP values in $MoS_{2}$ with different surface stoichiometries. The scanning electron microscopy (SEM) results showed that single crystalline $MoS_{2}$ flakes exhibit high crystal quality and uniform thickness.

**Table A2:** Studies of liquid-phase exfoliation.

Author Name	Paper Title	Publication Year	Findings/Results
[31]	Underwater superoleophobic and underoil superhydrophobic surface made by liquid-exfoliated MoS_2_ for on-demand oil-water separation	2019	The study aimed to develop a novel approach for oil–water separation using atomically thin $MoS_{2}$ nanosheets with the help of mechanical exfoliation. It was noted that the special properties of the $MoS_{2}$-coated fabric, like exhibiting both underwater and underoil superoleophobicity, make it highly effective for industrial and environmental applications.
[32]	Liquid-Phase Exfoliation and Functionalization of MoS_2_ Nanosheets for Effective Antibacterial Application	2020	An effective antibacterial agent was developed by synthesizing thin sheets of $MoS_{2}$ with the help of lysozyme-assisted LPE. It was found that the method used for functionalizing the nanosheets of $MoS_{2}$ resulted in good physiological stability.
[33]	Production and Patterning of Liquid Phase-Exfoliated 2D Sheets for Applications in Optoelectronics Production and Patterning of Liquid Phase-Exfoliated 2D Sheets for Applications	2019	A study for the production and patterning of high-quality 2D materials highlighted the ability to tune the properties of two-dimensional materials by manipulating their structure at the atomic level.
[34]	A MoS_2_ nanosheet-based CRISPR/Cas12a biosensor for efficient miRNA quantification for acute myocardial infarction	2024	A highly sensitive, antibody-free method was developed for the detection of miR-499 with the help of $MoS_{2}$ nanosheets combined with the CRISPR system.

**Table A3:** Studies of chemical vapor deposition method.

Author Name	Paper Title	Publication Year	Findings/Results
[41]	Ambient Pressure Chemical Vapor Deposition of Flat and Vertically Aligned MoS_2_ Nanosheets	2022	Ambient Pressure Chemical Vapor Deposition (AP-CVD) was used to synthesize thin nanosheets of molybdenum disulfide by using sulfur and molybdenum trioxide as precursor materials. The temperature and position of precursors are controlled by utilizing a two-zone furnace setup. Their findings recorded the importance and versatility of the AP-CVD method for producing high-quality thin $MoS_{2}$ nanosheets.
[42]	Capping technique for chemical vapor deposition of large and uniform MoS_2_ flakes	2021	Another method for the growth of thin nanosheets using CVD has been where two pieces of substrates are taken and cleaned. One piece of the substrate undergoes spin-coating with $Na_{2}MoO_{2}$ solution and the other substrate is placed on the top of the first substrate to create a sandwich structure. This prepared substrate is placed inside a quartz tube which is heated at 150 °C for 1 h along with sulfur in an alumina boat. This process leads to the formation of $MoS_{2}$ flakes on both substrates simultaneously, resulting in a uniform and high-quality monolayer of $MoS_{2}$ flakes on the substrate.
[43]	Effect of Growth Temperature on Physical Properties of MoS_2_ Thin Films Synthesized by CVD	2020	Thin nanosheets are grown using CVD where two pieces of substrates are taken and cleaned. One piece of the substrate undergoes spin-coating with $Na_{2}MoO_{2}$ solution and the other substrate is placed on the top of the first substrate to create a sandwich structure. This prepared substrate is placed inside a quartz tube which is heated at 150 °C for 1 h along with sulfur in an alumina boat. This process leads to the formation of $MoS_{2}$ flakes on both substrates simultaneously, resulting in a uniform and high-quality monolayer of $MoS_{2}$ flakes on the substrate.
[44]	Stepwise Sulfurization from MoO_3_ to MoS_2_ via Chemical Vapor Deposition	2018	A systematic investigation of the CVD growth of $MoS_{2}$ is presented, which mainly focuses on the effect of sulfur concentration. It was found that controlling sulfur vapor concentration is crucial to achieving high-quality $MoS_{2}$ through the CVD process, hence providing a deep understanding of the growth mechanism and potential for optimizing the synthesis of $MoS_{2}$ and other transition metal dichalcogenides.
[45]	Edge-Enriched 2D MoS_2_ Thin Films Grown by Chemical Vapor Deposition for Enhanced Catalytic Performance	2017	The researchers employed CVD to grow thin films of edge-enriched 2D $MoS_{2}$ with a nanoplatelet network structure and were successful in tailoring the CVD parameters to create a high-porosity network of $MoS_{2}$, where large platelets of $MoS_{2}$ had small layered $MoS_{2}$ sheets perpendicular to them, which significantly increased the number of edge sites within a given geometric area. It was also identified that increasing the thickness of the film leads to an increase in impedance and reduced current density. Their findings highlighted the potential of their edge-enriched $MoS_{2}$ films for catalytic applications, particularly in hydrogen production through water splitting.

**Table A4:** Studies of chemical exfoliation method.

Author Name	Paper Title	Publication Year	Findings/Results
[56]	Near-infrared photodetector achieved by chemically-exfoliated multilayered MoS_2_ flakes	2018	A device was developed with the help of chemically exfoliated multilayered $MoS_{2}$ films, where some amount of $MoS_{2}$ was dissolved in 16.5 M of butyllithium solution for about 47–48 h in a nitrogen-filled flask. Further, ultrasonication was performed for about 1 h, followed by centrifugation to remove all the excess unexfoliated materials. Vacuum filtration method was used to create $MoS_{2}$ films on silicon substrate.
[57]	Synthesis, characterization and photocatalytic performance of chemically exfoliated MoS_2_	2018	A study aimed to focus on the synthesization of $MoS_{2}$-based nanosheets used for photocatalytic applications. In the study, $MoS_{2}$ nanosheets were synthesized with the help of one-plot hydrothermal method by exfoliating them in dimethylformamide solution. The method presented by the study produced $MoS_{2}$ nanosheets with enhanced photocatalytic performance, providing a promising material for environmental applications such as pollutant degradation.
[58]	Robust Photodetectable Paper from Chemically Exfoliated MoS_2_-MoO_3_ Multilayers	2019	The performance of a flexible photodetector was investigated based on molybdenum disulfide and trioxide mixture, where powder of $MoS_{2}$ was chemically exfoliated with the help of n-butyllithium. The researchers were successful in manufacturing the flexible photodetector using a mixture of $MoS_{2}$ and $MoS_{3}$, resulting in a device with a high quantum efficiency.
[59]	Cost effective liquid phase exfoliation of MoS_2_ nanosheets and photocatalytic activity for wastewater treatment enforced by visible light	2020	An environmentally friendly method was developed to synthesize few-layer $MoS_{2}$ nanosheets using acetone as a solvent because of its low boiling point, low toxicity, and surface tension energy instead of using NMP. The thin nanosheets were prepared in various initial concentrations of bulk $MoS_{2}$ (0.08 mg/mL, 0.12 mg/mL, 0.3 mg/mL, and 0.4 mg/mL) with the help of an ultrasonic bath. To achieve the stable few-layered $MoS_{2}$ nanosheets, sonication time, duration of centrifugation, and amount of solvent were varied.

**Table A5:** Studies of physical vapor deposition method.

Author Name	Paper Title	Publication Year	Findings/Results
[64]	Optical and morphological properties of environmentally benign Cu-Tin sulfide thin films grown by physical vapor deposition technique	2019	PVD was used to synthesize copper-doped and undoped thin films of tin sulfide. Their findings showed that doping the thin films of SnS with copper leads to the alteration of the optical and structural properties.
[65]	Sputter deposition of 2D MoS_2_ thin films—A critical review from a surface and structural perspective	2022	The PVD process takes place inside a vacuum chamber to create thin films of $MoS_{2}$ on different substrates. In this process, $MoS_{2}$ is bombarded with high-energy argon ions, which causes the molecules to detach and deposit onto a substrate to form a thin film. This approach leads to a scalable and controlled fabrication of high-quality $MoS_{2}$ thin nanosheets.
[66]	Surface plasmon resonance biosensor sensitivity improvement employing of 2D materials and BaTiO_3_ with bimetallic layers of silver	2023	A surface plasmon resonance biosensor was fabricated, which was built on the bimetallic layers of Ag and MoS_2_ using the ATR method. The biosensor was fabricated on the BK7 prism base, and a layer of Ag was deposited on the BK7 base using the PVD method, followed by the formation of other layers using the CVD method. The authors revealed that the proposed biosensor tends to have much better sensitivity than the other conventional structures.
[67]	Efficient Point-of-Care Detection of Uric Acid in the Human Blood Sample with an Enhanced Electrocatalytic Response Using Nanocomposites of Cobalt and Mixed-Valent Molybdenum Sulfide	2022	A method was employed to detect uric acid in human blood using $MoS_{2}$ along with some nanocomposites of cobalt on a copper substrate with the help of PVD approach. It was recorded that the fabricated device has improved sensitivity, a long shelf life of about six months, and high specificity, which make it suitable for real-time clinical applications.
[68]	Chemical Vapor Deposition Synthesis Of Graphene And Transition Metal Dichalcogenides And Their Applications	2023	The physical vapor deposition method was employed to fabricate graphene-based field-effect transistors. PVD was used for the deposition of metal contacts on graphene during the fabrication process of GFETs.
